# Modeling membrane nanotube morphology: the role of heterogeneity in composition and material properties

**DOI:** 10.1038/s41598-020-59221-x

**Published:** 2020-02-13

**Authors:** Haleh Alimohamadi, Ben Ovryn, Padmini Rangamani

**Affiliations:** 1 Department of Mechanical and Aerospace Engineering, University of California San Diego, San Diego, CA 92093 USA; 2 Department of Physics, New York Institute of Technology, New York, NY 11568 USA

**Keywords:** Computational biophysics, Membrane biophysics

## Abstract

Membrane nanotubes are dynamic structures that may connect cells over long distances. Nanotubes are typically thin cylindrical tubes, but they may occasionally have a beaded architecture along the tube. In this paper, we study the role of membrane mechanics in governing the architecture of these tubes and show that the formation of bead-like structures along the nanotubes can result from local heterogeneities in the membrane either due to protein aggregation or due to membrane composition. We present numerical results that predict how membrane properties, protein density, and local tension compete to create a phase space that governs the morphology of a nanotube. We also find that there exists a discontinuity in the energy that impedes two beads from fusing. These results suggest that the membrane-protein interaction, membrane composition, and membrane tension closely govern the tube radius, number of beads, and the bead morphology.

## Introduction

**Figure 1:**
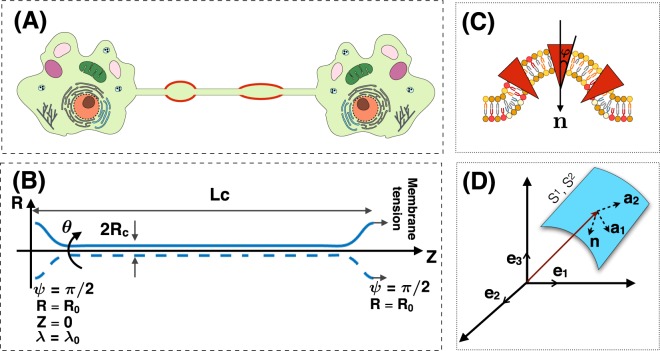
(**A**) A cartoon showing an intercellular membrane nanotube with local bead-shaped deformations due to membrane-protein interactions (red domain). (**B**) Axisymmetric coordinates along the membrane nanotube and the boundary conditions used in simulations. L_c_ represents the length and R_c_ represents the radius of the nanotube. (**C**) A schematic depicting membrane-protein interactions that could lead to the formation of beads along a nanotube. Proteins (shown in red) can aggregate along the membrane to induce local curvature and heterogeneous tension. We assume these proteins are cone-shaped such that their meridian makes an angle *φ* (*φ* < 0) with the normal vector (**n**) to the surface. (**D**) The coordinate system used to define a surface by the tangent basis **a**_1_, **a**_2_ and the normal vector **n**. Note that the normal is pointing downwards in this case.

Membrane nanotubes also known as tunneling nanotubes, have been identified as intercellular structures that can connect cells over long distances, i.e., tens of micrometers (see Fig. 1A)^1,2^. Membrane nanotubes have been observed in a wide variety of cell types^2–5^ in addition to the artificial nanotubes that have been produced from lipid vesicles^2,6^. Nanotubes are typically long and thin cylindrical protrusions with sub-micron diameter and lengths on the order of several hundred microns^1^. In contrast to other types of cellular projections, such as filopodia, which are attached to the substrate^7–9^, nanotubes are suspended in the medium^5,10–13^. Despite increasing observations in the literature highlighting the functional role of membrane nanotubes, the role of membrane mechanics in governing the morphology of these structures has largely remained unexplored.

Changes in the surrounding environment, rearrangement of membrane constituents, or mechanical stresses can result in dramatic shape transformations^1,14–18^. For instance, in addition to the predominantly cylindrical geometry of many membrane nanotubes, the formation of pearl shapes along membrane nanotubes has been observed in various experiments^12,19–21^. This morphological transition in nanotube structures resembles the well-known Rayleigh-Plateau fluid instability, where a change in the surface tension results in the break up of a cylindrical stream of fluid into multiple droplets^22–24^. Similarly, in the case of cylindrical membranes, various studies have shown a tension-driven Rayleigh-type instability, in which a perturbation in the membrane tension leads to the pearling instability^25–30^. Indeed, the formation of beads along membrane nanotubes could result from any asymmetry in the lipid bilayer such as protein-induced spontaneous curvature arising from local phase separation of proteins, adsorption of nanoparticles and/or anchored polymers, or induced anisotropic curvatures by membrane inclusions^15,31–33^. These interactions are accompanied by a change in the local tension of the membrane^34–38^.

A series of elegant modeling studies have proposed the idea that curved proteins and cytoskeletal proteins can induce protrusions along the membrane^39–43^. Separately, experiments are have demonstrated that: (a) the composition of a membrane nanotube is not homogeneous^44^; (b) tension due to adhesion of rolling neutrophils can lead to tether formation^44^; and (c) spontaneous curvature along a nanotube can lead to the formation of bead-like structures^17,20,44–47^. It is not yet clear if such beaded structures are common to membrane nanotubes and if they have a physiological role in cells and tissues. Nonetheless, from a membrane mechanics standpoint, these structures are fascinating to study. Here, we examine how changes in the local membrane tension, originating from the heterogeneous distribution of the membrane components due to local phase separation, can cause the shape transformation from a cylindrical membrane to a beaded architecture. Therefore, we seek to answer the following questions: how does the presence of a membrane protein domain affect the shape of a nanotube? What is the phase space that governs the energy landscape of membrane nanotubes? And finally, how do multiple beads interact with each other along the surface of a nanotube? To address these questions, we conducted simulations by implementing an augmented Helfrich model that includes protein density contributions^48–51^ with local incompressibility constraints^36,52^.

## Methods

### Assumptions

The plasma membrane is a complex structure; various molecules pack tightly together to form a semi-impermeable barrier for the cytoplasm, nucleus, and intercellular organelles^53^. Nevertheless, under certain assumptions as described below, it is appropriate to model this complex and heterogeneous surface using a simple mathematical framework.

- The length scale of the nanotube is assumed to be much larger (~20 times) than the thickness of the bilayer such that the membrane behaves as a thin elastic shell^51,54,55^.
- We assume that the membrane nanotube is at mechanical equilibrium (i.e. no inertia)^56^. Because of the high stretching modulus of lipid bilayers^57^, we also assume that the lipid bilayer is areally incompressible and we use a Lagrange multiplier to implement this constraint^55,58,59^.
- We consider the case that there is a local phase separation of proteins along the membrane surface (see Fig. 1A). We assume that the local phase separated membrane proteins are rotationally symmetric, induce a negative spontaneous curvature ($\varphi  < 0$), and ignore the influence of anisotropic curvature inducing proteins such as BIN-Amphiphysin-Rvs (BAR) domain proteins^40,41,60^.
- We assume that the total energy of the system includes the membrane bending energy and a contribution from local protein phase separation in a dilute regime (low protein density). Thus, the membrane energy is modeled using an augmented version of Helfrich energy for elastic manifolds including membrane-protein interaction contributions^61–66^. In mechanical equilibrium, the total energy of the membrane including the effects of the local phase separation of proteins is similar to a standard Cahn-Hilliard model^67^ with the gradient penalty for the spatial heterogeneity of the protein distribution. For simplicity, we prescribe the local distribution of proteins along the surface of the membrane nanotube using a hyperbolic tangent function (Eq. S33)^68–70^.
- We do not consider the role of any other forces, e.g. actin^40^, so that we can focus only on membrane nanotube deformation due to membrane-protein interactions^71–73^.
- For simplicity in the numerical simulations, we assume that the membrane in the region of interest is rotationally symmetric and long enough so that boundary effects can be ignored (Fig. 1B)^31,73^.

### Membrane energy and equations of motion

We model the membrane with two contributions to the strain energy – one from protein-protein interactions and the other from the membrane bending. The protein-protein interaction is written as a constitutive function of the protein density *σ* (number per unit area). While the exact form of this energy is yet to be experimentally verified, based on thermodynamic arguments, the dependence of the energy on the local protein density, the protein density gradient, and the thermal entropic contribution has been proposed as^64,66,74–77^

1 $${W}_{{\rm{Protein}}}=\mathop{\underbrace{-\alpha \sigma {({\theta }^{\xi })}^{2}}}\limits_{{\rm{Protein}}\,{\rm{aggregation}}}+\mathop{\underbrace{\beta {(\nabla \sigma )}^{2}}}\limits_{{\rm{Inhomogeneous}}\,{\rm{protein}}\,{\rm{distribution}}}+\mathop{\underbrace{{k}_{B}T\sigma (\log (\frac{\sigma }{{\sigma }_{s}})-1)}}\limits_{{\rm{Entropic}}\,{\rm{contribution}}\,{\rm{due}}\,{\rm{to}}\,{\rm{thermal}}\,{\rm{diffusion}}},$$

where $W$ is the energy per unit area, $\alpha$ indicates the strength of the attractive energy between two neighboring proteins, $\beta$ is a positive constant that depends on the excluded area and the effective interaction area of the proteins^67,78^, $\nabla$ is the gradient operator, ${k}_{B}$ is the Boltzmann constant, and *T* is temperature^64,75^. In Eq. 1, $\beta {(\nabla \sigma )}^{2}$ is a first order correction for a spatial inhomogeneity in the membrane composition that allows the interfacial energy to be modeled with a sharp gradient in a continuous surface^67^. We should mention that in Eq. 1, we ignore the higher order terms in *σ* ^66,76^ since we assume that the system is in the dilute regime and the protein density is low.

In Eq. 1, $\sigma$ can depend explicitly on the surface coordinates ${\theta }^{\xi }$ to allow for local heterogeneity (Fig. 1D). Also, the proteins are assumed to be transmembrane, conical insertions such that the meridian of each protein is at an angle $\varphi$ ($\varphi  < 0$) with the normal vector to the membrane surface (**n**) (Fig. 1C)^49^. In the dilute regime, the locally induced-spontaneous curvature due to membrane-protein interaction can be modeled as a linear function of the surface protein density as^49^

2 $$C(\sigma )=\mu \varphi \sigma ({\theta }^{\xi }),$$

where *μ* is a length scale that represents the lipid-protein specific entropic interactions. The energy associated with membrane bending due to the isotropic spontaneous curvature is given by the Helfrich Hamiltonian, modified to include the heterogeneous membrane properties as^36,51,79,80^

3 $${W}_{{\rm{Bending}}}=\kappa ({\theta }^{\xi }){[H-C(\sigma ({\theta }^{\xi }))]}^{2}+{\kappa }_{G}({\theta }^{\xi })K,$$

where H is the local mean curvature and K is the local Gaussian curvature. $\kappa$ and ${\kappa }_{G}$ are bending and Gaussian moduli respectively, which can vary along the surface coordinate ${\theta }^{\xi }$ ^36,79,81^. Hence, the total energy of the membrane including both bending and protein contributions is given by

4 $$\begin{array}{rcl}W(H,K,\sigma ;{\theta }^{\xi }) & = & \mathop{\underbrace{\kappa ({\theta }^{\xi }){[H-C(\sigma ({\theta }^{\xi }))]}^{2}+{\kappa }_{G}({\theta }^{\xi })K}}\limits_{{\rm{Bending}}}\mathop{\underbrace{-\alpha \sigma {({\theta }^{\xi })}^{2}}}\limits_{{\rm{Protein}}\,{\rm{aggregation}}}\\  &  & +\,\mathop{\underbrace{\beta {(\nabla \sigma )}^{2}}}\limits_{{\rm{Inhomogeneous}}\,{\rm{protein}}\,{\rm{distribution}}}+\mathop{\underbrace{{k}_{B}T\sigma (\log (\frac{\sigma }{{\sigma }_{s}})-1)}}\limits_{{\rm{Entropic}}\,{\rm{contribution}}\,{\rm{due}}\,{\rm{to}}\,{\rm{thermal}}\,{\rm{diffusion}}}.\end{array}$$

We note that $\frac{{k}_{B}T}{\kappa }$ is small because the membrane bending modulus is in the range of 20–40 ${k}_{B}T$ ^82,83^. Additionally, in the dilute regime of low protein density ${k}_{B}T\sigma \ll 1$. Based on this analysis, we neglect the entropic term in the rest of our calculations.

A local balance of forces normal to the membrane, subject to the energy density given in Eq. (4) yields the so-called “shape equation”^49,50,84^

5 $$\begin{array}{c}\mathop{\underbrace{\Delta [\kappa (H-(\mu \varphi )\sigma )]-{({\kappa }_{G})}_{;\xi \eta }{\tilde{b}}^{\xi \eta }-2\kappa H{(H-(\mu \varphi )\sigma )}^{2}+2\kappa (H-(\mu \varphi )\sigma )(2{H}^{2}-K)}}\limits_{{\rm{Elastic}}\,{\rm{effects}}}\\ +\,\mathop{\underbrace{2H(\alpha {\sigma }^{2})}}\limits_{{\rm{Protein}}\,{\rm{aggregation}}}-\mathop{\underbrace{2H\beta {(\nabla \sigma )}^{2})}}\limits_{{\rm{Inhomogeneous}}\,{\rm{protein}}\,{\rm{distribution}}}=\mathop{\underbrace{(p+2\lambda H)}}\limits_{{\rm{Capillary}}\,{\rm{effect}}},\end{array}$$

where $\Delta$ is the surface Laplacian operator, $p$ is the pressure difference across the membrane, $\lambda$ is interpreted as the membrane tension^36,55^, (); is the covariant derivative with the respect to the surface metric, and ${\tilde{b}}^{\xi \eta }$ is the co-factor of the curvature tensor. A local balance of forces tangent to the membrane, which enforces the incompressibility condition in a heterogeneous membrane, yields the spatial variation of membrane tension *λ*^36,49,52,85^,

6 $$\begin{array}{c}\mathop{\underbrace{{\rm{\nabla }}\lambda }}\limits_{{\rm{G}}{\rm{r}}{\rm{a}}{\rm{d}}{\rm{i}}{\rm{e}}{\rm{n}}{\rm{t}}\,{\rm{o}}{\rm{f}}\,{\rm{m}}{\rm{e}}{\rm{m}}{\rm{b}}{\rm{r}}{\rm{a}}{\rm{n}}{\rm{e}}\,{\rm{t}}{\rm{e}}{\rm{n}}{\rm{s}}{\rm{i}}{\rm{o}}{\rm{n}}}\,=\,\mathop{\underbrace{2[\kappa \mu \varphi (H-(\mu \varphi )\sigma )+\alpha ]\frac{{\rm{\partial }}\sigma }{{\rm{\partial }}{\theta }^{\xi }}-\beta ({\rm{\nabla }}\sigma )\frac{{\rm{\partial }}({\rm{\nabla }}\sigma )}{{\rm{\partial }}{\theta }^{\xi }}}}\limits_{{\rm{P}}{\rm{r}}{\rm{o}}{\rm{t}}{\rm{e}}{\rm{i}}{\rm{n}}\,{\rm{d}}{\rm{e}}{\rm{n}}{\rm{s}}{\rm{i}}{\rm{t}}{\rm{y}}\,{\rm{v}}{\rm{a}}{\rm{r}}{\rm{i}}{\rm{a}}{\rm{t}}{\rm{i}}{\rm{o}}{\rm{n}}}\\ -\mathop{\underbrace{\frac{{\rm{\partial }}\kappa }{{\rm{\partial }}{\theta }^{\xi }}{(H-(\mu \varphi )\sigma )}^{2}}}\limits_{{\rm{B}}{\rm{e}}{\rm{n}}{\rm{d}}{\rm{i}}{\rm{n}}{\rm{g}}\,{\rm{m}}{\rm{o}}{\rm{d}}{\rm{u}}{\rm{l}}{\rm{u}}{\rm{s}}-{\rm{i}}{\rm{n}}{\rm{d}}{\rm{u}}{\rm{c}}{\rm{e}}{\rm{d}}\,{\rm{v}}{\rm{a}}{\rm{r}}{\rm{i}}{\rm{a}}{\rm{t}}{\rm{i}}{\rm{o}}{\rm{n}}}-\mathop{\underbrace{\frac{{\rm{\partial }}{\kappa }_{G}}{{\rm{\partial }}{\theta }^{\xi }}K}}\limits_{{\rm{G}}{\rm{a}}{\rm{u}}{\rm{s}}{\rm{s}}{\rm{i}}{\rm{a}}{\rm{n}}\,{\rm{m}}{\rm{o}}{\rm{d}}{\rm{u}}{\rm{l}}{\rm{u}}{\rm{s}}-{\rm{i}}{\rm{n}}{\rm{d}}{\rm{u}}{\rm{c}}{\rm{e}}{\rm{d}}\,{\rm{v}}{\rm{a}}{\rm{r}}{\rm{i}}{\rm{a}}{\rm{t}}{\rm{i}}{\rm{o}}{\rm{n}}}.\end{array}$$

For the sake of brevity, the specialization of the governing equations to axisymmetric coordinates is provided in the SOM along with tables of notation and parameter values (Tables S1–S3).

### Analytical solutions (limit cases)

In this section, we explore the analytical solution for Eq. 4, ignoring the boundary effects, the Gaussian curvature, and the entropic term. In this condition, for a nanotube with uniform bending rigidity and no protein density, the free energy density (Eq. 4) is given by ${W}_{0}=\kappa {H}_{0}^{2}$, where ${H}_{0}$ is the mean curvature of the nanotube equal to $1/(2{{\rm{R}}}_{{\rm{c}}})$ (${H}_{0}=1/(2{{\rm{R}}}_{{\rm{c}}})$). ${W}_{0}$ is the energy minimizer for this geometry; adding proteins locally or including heterogeneous bending rigidity increases the energy of the system ($W\ge {W}_{0}$) (see Eq. S28). To find an analytical expression for the mean curvature of the nanotube at the center of the protein-enriched domain as a function of the protein density and the bending rigidity, we consider the limit case that $W={W}_{0}$. This gives us an expression for the mean curvature at the center of protein-enriched domain as

7 $$\begin{array}{l}{H}_{{\rm{analytical}}}=\mathop{\underbrace{\mu \varphi \sigma }}\limits_{{\rm{Spontaneous}}\,{\rm{curvature}}}+\sqrt{\mathop{\underbrace{\frac{\frac{1}{{(2{R}_{{\rm{c}}})}^{2}}}{{\kappa }_{{\rm{ratio}}}}}}\limits_{{\rm{Preexisting}}\,{\rm{curvature}}\,{\rm{of}}\,{\rm{the}}\,{\rm{tube}}}+\mathop{\underbrace{\frac{\frac{\alpha {\sigma }^{2}}{\kappa }}{{\kappa }_{{\rm{ratio}}}}}}\limits_{{\rm{Aggregation}}\,{\rm{effects}}}-\mathop{\underbrace{\frac{\frac{\beta {(\nabla \sigma )}^{2}}{\kappa }}{{\kappa }_{{\rm{ratio}}}}}}\limits_{{\rm{Inhomogeneous}}\,{\rm{protein}}\,{\rm{distribution}}}},\end{array}$$

Here, ${\kappa }_{{\rm{ratio}}}$ represents the ratio of the bending rigidity in the protein-enriched domain (${\kappa }_{{\rm{protein}}}$) compared to the bending rigidity of the bare lipid membrane (${\kappa }_{{\rm{ratio}}}={\kappa }_{{\rm{protein}}}/\kappa$).

For low protein density ($\sigma \ll 1$), the higher order terms in Eq. 7 can be ignored and the equation can be simplified using Taylor expansion around *σ* which gives

8 $$\begin{array}{ccc}{H}_{{\rm{a}}{\rm{n}}{\rm{a}}{\rm{l}}{\rm{y}}{\rm{t}}{\rm{i}}{\rm{c}}{\rm{a}}{\rm{l}}} & = & \mathop{\underbrace{\mu \varphi \sigma }}\limits_{{\rm{S}}{\rm{p}}{\rm{o}}{\rm{n}}{\rm{t}}{\rm{a}}{\rm{n}}{\rm{e}}{\rm{o}}{\rm{u}}{\rm{s}}\,{\rm{s}}{\rm{c}}{\rm{u}}{\rm{r}}{\rm{v}}{\rm{a}}{\rm{t}}{\rm{u}}{\rm{r}}{\rm{e}}}+\frac{1}{\sqrt{{\kappa }_{{\rm{r}}{\rm{a}}{\rm{t}}{\rm{i}}{\rm{o}}}}}(\mathop{\underbrace{\frac{1}{(2{R}_{{\rm{c}}})}}}\limits_{{\rm{P}}{\rm{r}}{\rm{e}}{\rm{e}}{\rm{x}}{\rm{i}}{\rm{s}}{\rm{t}}{\rm{i}}{\rm{n}}{\rm{g}}\,{\rm{c}}{\rm{u}}{\rm{r}}{\rm{v}}{\rm{a}}{\rm{t}}{\rm{u}}{\rm{r}}{\rm{e}}\,{\rm{o}}{\rm{f}}\,{\rm{t}}{\rm{h}}{\rm{e}}\,{\rm{t}}{\rm{u}}{\rm{b}}{\rm{e}}}\\  &  & +\frac{{R}_{{\rm{c}}}}{\kappa }(\mathop{\underbrace{\alpha {\sigma }^{2}}}\limits_{{\rm{A}}{\rm{g}}{\rm{g}}{\rm{r}}{\rm{e}}{\rm{g}}{\rm{a}}{\rm{t}}{\rm{i}}{\rm{o}}{\rm{n}}\,{\rm{e}}{\rm{f}}{\rm{f}}{\rm{e}}{\rm{c}}{\rm{t}}{\rm{s}}}-\mathop{\underbrace{\beta {({\rm{\nabla }}\sigma )}^{2}}}\limits_{{\rm{I}}{\rm{n}}{\rm{h}}{\rm{o}}{\rm{m}}{\rm{o}}{\rm{g}}{\rm{e}}{\rm{n}}{\rm{e}}{\rm{o}}{\rm{u}}{\rm{s}}\,{\rm{p}}{\rm{r}}{\rm{o}}{\rm{t}}{\rm{e}}{\rm{i}}{\rm{n}}\,{\rm{d}}{\rm{i}}{\rm{s}}{\rm{t}}{\rm{r}}{\rm{i}}{\rm{b}}{\rm{u}}{\rm{t}}{\rm{i}}{\rm{o}}{\rm{n}}})).\end{array}$$

**Figure 2:**
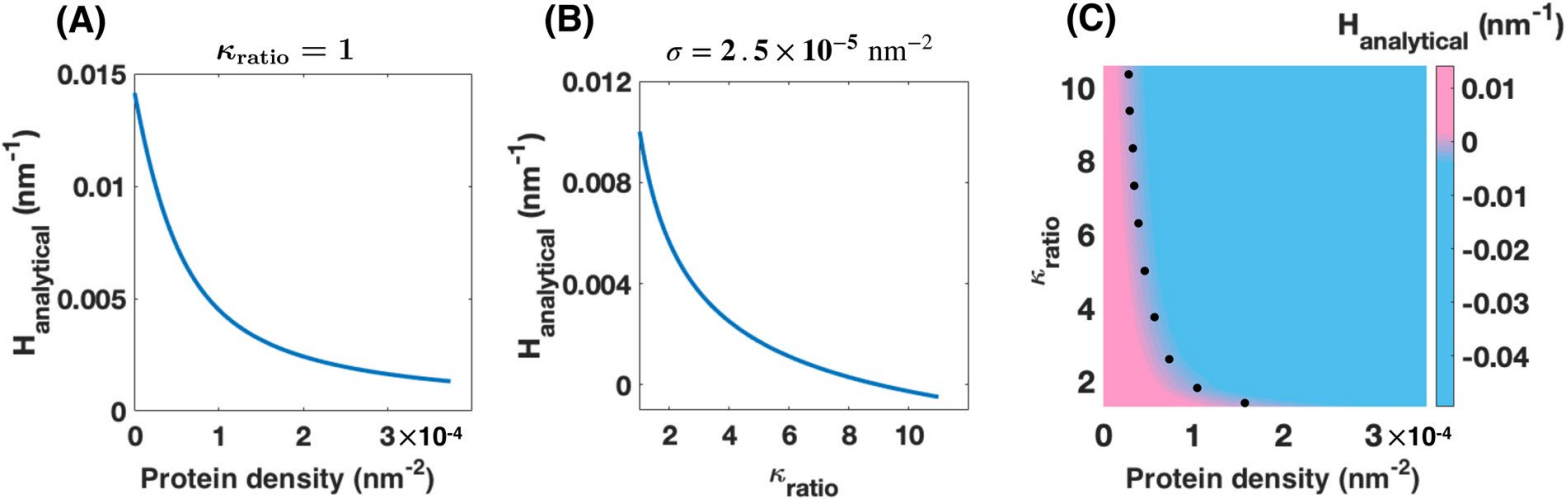
Analytical mean curvature along the protein-enriched (Eq. 7) domain as a function of the protein density (*σ*) and bending rigidity ratio (${\kappa }_{{\rm{ratio}}}$). (**A**) With increasing the protein density, the mean curvature along the protein aggregation domain decreases (${\kappa }_{{\rm{ratio}}}=1$). (**B**) Decrease in the mean curvature of the protein-enriched domain as the bending rigidity ratio increases ($\sigma =2.5\times {10}^{-5}\,{{\rm{nm}}}^{-5}$). (**C**) Heat map shows the analytical mean curvature along the protein-enriched domain (Eq. 8) as a function of the protein density and bending rigidity ratio. The sign of the analytical mean curvature changes from positive to negative along the dotted black line.

The relationship in Eq. 7 can be used to predict how the local curvature of the nanotube at the center of the protein-enriched domain varies with protein density and ${\kappa }_{{\rm{ratio}}}$. For a homogenous membrane (${\kappa }_{{\rm{ratio}}}=1$), as *σ* increases, the negative spontaneous curvature becomes dominant and the mean curvature at the center of the protein-enriched domain decreases (Fig. 2A). For a constant protein density ($\sigma =2.5\times {10}^{-5}\,{{\rm{nm}}}^{-2}$), as ${\kappa }_{{\rm{ratio}}}$ increases, the positive term under the square root becomes smaller and therefore the mean curvature decreases (Fig. 2B). A decrease in the mean curvature of the nanotube at the center of the protein-enriched domain corresponds to a larger radius in that point (Eq. S20). This implies that protein aggregation with heterogeneous properties alters the morphology of the membrane nanotube where bead-shaped structures with larger radii than the nanotube radius (${{\rm{r}}}_{{\rm{b}}} > {{\rm{R}}}_{{\rm{c}}}$, where *r*_*b*_ is the radius of the bead) forms along the protein-enriched domains.

In Fig. 2C, we plotted the derived analytical mean curvature at the center of the protein-enriched domain (Eq. 7) as a function of protein density and bending rigidity ratio. Interestingly, we observed that the sign of the analytical mean curvature at the center of the protein-enriched domain changes from positive (pink domain) to negative (blue domain) for large values of the bending rigidity ratio and protein density. However, we know that for a rotational symmetric nanotube, the mean curvature at the center of the protein-enriched domain cannot be negative. This suggests that for low protein density and bending rigidity ratio (pink domain in Fig. 2C), the energy of the system can be minimized with decreasing the local mean curvature along the protein-enriched domain. But, for large protein density and the bending rigidity ratio (blue domain in Fig. 2C), the system needs to exploit another mechanism to lower the work done on the system.

We next relate the analytical curvature in the protein-enriched domain (Eq. 7) to the radius of the bead assuming that the bead shape can be approximated by a cylinder and therefore ${H}_{{\rm{analytical}}}=1/(2{{\rm{r}}}_{{\rm{b}},{\rm{analytical}}})$. Thus, using Eq. 7, we have

9 $$\begin{array}{l}2{{\rm{r}}}_{{\rm{b}},{\rm{analytical}}}={[\mu \varphi \sigma +\sqrt{\frac{\frac{1}{{(2{{\rm{R}}}_{c})}^{2}}+\frac{\alpha {\sigma }^{2}}{\kappa }-\frac{\beta {(\nabla \sigma )}^{2}}{\kappa }}{{\kappa }_{{\rm{ratio}}}}}]}^{-1}.\end{array}$$

Similar to Eq. 7, we can simplify Eq. 9 for low protein density given by

10 $$\begin{array}{l}{{\rm{r}}}_{{\rm{b}},{\rm{analytical}}}=\sqrt{{\kappa }_{{\rm{ratio}}}}{R}_{{\rm{c}}}(1-2{R}_{{\rm{c}}}\sqrt{{\kappa }_{{\rm{ratio}}}}(\mu \varphi \sigma )-\frac{2{R}_{{\rm{c}}}^{2}}{\kappa }(\alpha {\sigma }^{2}-\beta {(\nabla \sigma )}^{2})).\end{array}$$

Because Eq. 10 is valid only for small membrane deformation, we use the mechanical model (Eqs. 5 and 6) to run simulations and obtain the shape and the size of the large beads.

### Numerical implementation

In axisymmetric coordinates, the equations of motion (Eqs. S14 and S15) simplify to a system of first-order differential equations (Eq. S24) with six prescribed boundary conditions (Eq. S25). In order to solve these coupled equations, we used the commercially available finite element solver COMSOL MULTIPHYSICS. In this work, we assume that the total length of the membrane nanotube is conserved and to focus on the net effect of membrane tension, we set the transmembrane pressure at zero ($p=0$). The values of parameters used in the model are summarized in Table 1. All COMSOL files are available at http://www.github.com/Rangamani-Lab/Rangamni-Nanotubes2020 for public dissemination.

**Table 1:** Parameters used in the model.

Notation	Description	Value
*λ*_0_	Edge membrane tension^114,115^	0–0.064 pN/nm
*κ*	Bending rigidity of the bare lipid membrane^83^	320 pN · nm
*κ*_protein_	Bending modulus of the rigid protein-enriched domain^89,116,117^	320–9600 pN · nm
*σ*	Protein density^118^	0–3.75 × 10^−4^ nm^−2^
*α*	Strength of protein-protein interactions^49,64,65,74,78^	128 × 10^5^ pN · nm^3^
*μ*	Constant length scale^49^	200 nm
*φ*	The cone-shaped protein angle^49^	−1
L_c_	Nanotube length	20 μm
R_c_	Nanotube radius	35 nm
*k*_*B*_*T*	Boltzmann energy	4.114 pN · nm
*p*	Transmembrane pressure	0 pN · nm^−2^

## Results

### Formation of beads along a nanotube due to protein-induced spontaneous curvature

**Figure 3:**
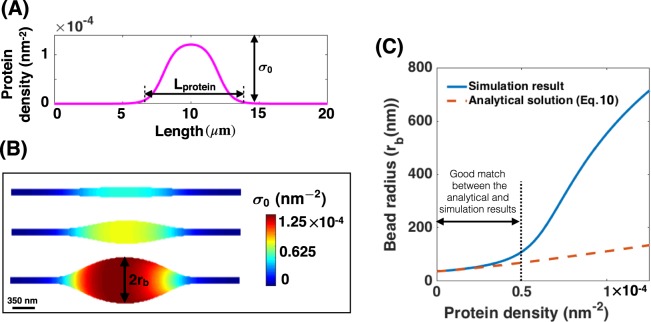
Protein-mediated bead formation along a membrane nanotube. (**A**) Protein density distribution on the membrane surface in which ${{\rm{L}}}_{{\rm{protein}}}=8\,\mu {\rm{m}}$ shows the length of the protein-enriched domain and *σ*_0_ represents the number of the proteins per unit area. (**B**) The formation of a large bead-shaped structure along the membrane nanotube as the density of proteins (*σ*_0_) increases for ${\lambda }_{0}=0.064\,\text{pN}/\text{nm}$ and uniform bending rigidity. The scale bar in panel (B) is 350 nm. (**C**) Bead radius (${{\rm{r}}}_{{\rm{b}}}$) increases as a function of the protein density for both the analytical solution (Eq. 10) (dashed red line) and the simulation result (solid blue line).

For membrane nanotubes, it has been shown that the composition of the lipid bilayer is a critical factor in determining their shapes and radii^29,73,86,87^. To explore how heterogeneity in the membrane properties due to a surface protein aggregation affects the shape of a nanotube, we conducted simulations on cylindrical nanotubes with the aspect ratio of radius R_c_ = 35  m and length L_c_ = 20 *μ*m, and set the boundary tension to ${\lambda }_{0}=0.064\,{\rm{pN}}/{\rm{nm}}$. The effect of boundary tension on the initial nanotube radius and length is shown in Fig. S1. To ensure a smooth continuous transition between the domains, we implemented the difference in the protein density using a hyperbolic tangent function (Eq. S33), such that the covered domain by the protein-enriched domain at the center of the nanotube remains constant (${{\rm{L}}}_{{\rm{protein}}}=8\,\mu {\rm{m}}$), and the number of proteins per area increases from ${\sigma }_{0}=0$ to ${\sigma }_{0}=1.25\times {10}^{-4}\,{{\rm{nm}}}^{-2}$ (Fig. 3A).

As the number of proteins in the fixed domain (${{\rm{L}}}_{{\rm{protein}}}$) is increased, the membrane bends outward such that it resembles a bead-shaped structure that forms along the nanotube (Fig. 3B). This bead formation can be understood by considering the energy of the membrane (Eq. 4). In the absence of any protein ($\sigma =0$, therefore $C=0$), the curvature of the nanotube is constant everywhere (${H}_{0}=1/(2{R}_{c})=1/70\,\mu {{\rm{m}}}^{-1}$). Increasing the density of the conical-shaped proteins induces a negative spontaneous curvature (see Fig. 1C) and therefore increases the bending energy (Eq. 4). As a result of this increase in energy, the membrane curvature locally decreases by bending outward in the domain of the protein aggregation to minimize the total energy (Fig. 3B). For example, with increasing the protein density from ${\sigma }_{0}=0$ to ${\sigma }_{0}=1.25\times {10}^{-4}\,{{\rm{nm}}}^{-2}$, a bead with a radius of ~350 m forms (Fig. 3B).

To understand the relationship between increasing *σ* and the shape of the bead, we compared the results from our simulations against the analytical approximation for the bead radius (Eq. 10) as a function of *σ* assuming a uniform bending rigidity all along the nanotube (${\kappa }_{{\rm{ratio}}}=1$) (Fig. 3C). For small protein densities (${\sigma }_{0} < 5\times {10}^{-5}\,{{\rm{nm}}}^{-2}$) and small membrane deformation (r_b_ < 200 m), we found a good match between the analytical approximation and numerical results (Fig. 3C). However, for larger protein densities, the membrane deformation along the protein-enriched domain is large and the bead no longer has a cylindrical shape anymore exposing the limits of the analytical expression.

We next investigated the effect of the extent of the protein aggregation in Eq. 1 on the bead morphology. We repeated the simulations in Fig. 3 for three different values of $\alpha$ (Fig. S2). We found that varying $\alpha$ does not alter the shape or the radius of the bead significantly (Fig. S2). This is because the protein aggregation term in the energy has a small contribution to membrane bending; the dominant effect comes from the coupling between the protein density and spontaneous curvature (Eqs. 2, 3).

Another important factor that controls the lipid flow on the surface of nanotubes and lipid packing is membrane tension^78,88^. Consistent with the previous studies^34–36^, we observed that a local decrease in the membrane tension of the beaded domain corresponding to membrane bending and the applied area incompressibility constraint (Fig. S3). This variation in the membrane tension allows us to also represent the radius of the bead radius as a function of the local membrane tension, where we found a nonlinear relationship between the increase in the radius of the bead and the decrease in the local membrane tension (Fig. S3).

### Heterogeneity in membrane stiffness lead to the formation of bead-like structures along a nanotube

**Figure 4:**
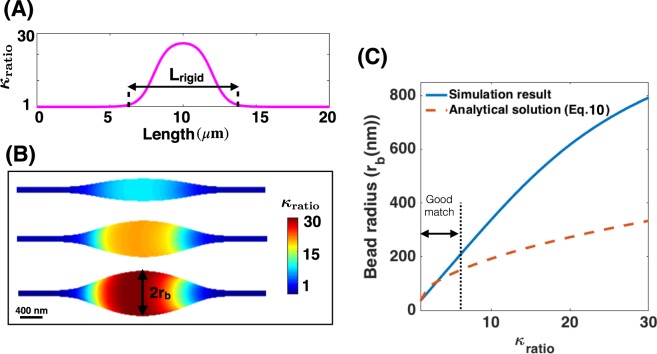
Heterogeneous membrane properties result in the formation of local bead-shaped structures. (**A**) Bending modulus variation along the length of the nanotube. ${\kappa }_{{\rm{ratio}}}$ is the bending rigidity ratio of the rigid protein domain compared to that of the bare lipid membrane (${\kappa }_{{\rm{ratio}}}={\kappa }_{{\rm{rigid}}}/{\kappa }_{{\rm{lipid}}}$) and ${{\rm{L}}}_{{\rm{rigid}}}$ represents the length of the rigid protein domain (${{\rm{L}}}_{{\rm{rigid}}}=8\,\mu {\rm{m}}$). (**B**) Membrane deformation in the region of large bending rigidity resembles a local bead formation phenomenon; the tension at the boundary is set as ${\lambda }_{0}=0.064\,{\rm{pN}}/{\rm{nm}}$ and the protein density is fixed to be constant as ${\sigma }_{0}=1.25\times {10}^{-4}\,{{\rm{nm}}}^{-2}$ and C = 0. The scale bar in panel (B) is 400 nm. (**C**) Increase in the radius of the bead as a function of ${\kappa }_{{\rm{ratio}}}$ for both the derived analytical solution in Eq. 10 (dashed red line) and the simulation result (solid blue line).

Motivated by our numerical observation that a protein-induced spontaneous curvature along the membrane can result in the bead-like structures, we next asked if a change in the membrane stiffness due to membrane-protein interaction could also induce a similar deformation along the nanotube. To answer this question, we repeated the simulation in Fig. 3 for ${\sigma }_{0}=1.25\times {10}^{-4}\,{{\rm{nm}}}^{-2}$ assuming that the bending rigidity along the domain covered by the proteins is higher than the rest of the membrane, but $C=0$ (e.g for cylindrical proteins where $\varphi =0$)^87,89^ (Fig. 4A). This represents a case where the membrane-protein interaction induces a change in the membrane composition but does not induce an asymmetry between the leaflets.

As it is clear, by increasing ${\kappa }_{{\rm{ratio}}}$ from ${\kappa }_{{\rm{ratio}}}=1$ to ${\kappa }_{{\rm{ratio}}}$ = 30^87,89,90^, the membrane bending energy (Eq. 4) increases. To compensate for this increase in the bending energy, the membrane curvature decreases by bending outward significantly in the domain of the rigid segment and an ellipsoidal bead-shaped structure forms along the nanotube (Fig. 4B). For instance, we found that with increasing bending rigidity ratio from ${\kappa }_{{\rm{ratio}}}=1$ to ${\kappa }_{{\rm{ratio}}}=30$, a large bead with a radius of ~400 nm forms in the domain of the rigid protein (Fig. 4B). Comparing radius of the bead obtained from numerical simulation (Fig. 4B) and the analytical expression (Eq. 10) as a function of the bending rigidity ratio for constant protein density (${\sigma }_{0}=1.25\times {10}^{-4}\,{{\rm{nm}}}^{-2}$), we observed that for low values of the bending rigidity ratio (${\kappa }_{{\rm{ratio}}} < 5$), the membrane deformation is small (${{\rm{r}}}_{{\rm{b}}} < 200\,{\rm{nm}}$) and therefore there is a good agreement between the analytical and simulation results (Fig. 4C). However, for large membrane deformations (when ${\kappa }_{{\rm{ratio}}} > 5$), the analytical solution underestimates the radius of the bead because of our assumption in Eq. 9 that the bead has a cylindrical shape is no longer valid (Fig. 4C).

Additionally, we observed a local reduction in the membrane tension with increasing the bending rigidity of the protein domain and the formation of the bead along the nanotube (Fig. S4). Interestingly, we found a similar trend in the reduction of the local membrane tension for both protein-induced spontaneous curvature and the protein rigidity mechanisms (Figs. S3 and S4). The membrane tension decreases from $\lambda =0.064\,{\rm{pN}}/{\rm{nm}}$ to about zero with the formation of a bead with a radius of ${{\rm{r}}}_{{\rm{b}}}=100\,{\rm{nm}}$. After that, for the larger beads, the local membrane tension is almost zero and remains constant (Figs. S3 and S4). For completeness, we also varied the Gaussian modulus along the domain of protein aggregation ($\Delta {\kappa }_{G}\,=\,({\kappa }_{G,{\rm{protein}}}-{\kappa }_{G,{\rm{lipid}}})/{\kappa }_{G,{\rm{lipid}}}$). Varying −20 < Δ${\kappa }_{G} < 20$ for ${\sigma }_{0}=1.25\times {10}^{-4}\,{{\rm{nm}}}^{-2}$ and ${\kappa }_{{\rm{ratio}}}=1$, we found that the changes in the Gaussian modulus alone lead to small membrane deformations as compared with other effects (see Fig. S5).

### Energy landscape of bead-shaped structures along a nanotube

Our simulation results have demonstrated that two unrelated mechanisms, protein-induced curvature, and heterogeneity in the membrane rigidity, each independently lead to the formation of the bead-like structures along the membrane nanotube and that the radius of the bead increases nonlinearly with increasing strength of the heterogeneity. In order to explore how these two mechanisms might interact and modulate the shape of a nanotube, we conducted simulations where the heterogeneous domain has effects from both the protein-induced spontaneous curvature and from increased bending rigidity. We repeated the simulations shown in Fig. 3 but this time assumed that the bending rigidity is heterogeneous (${\kappa }_{{\rm{ratio}}}=11$).

**Figure 5:**
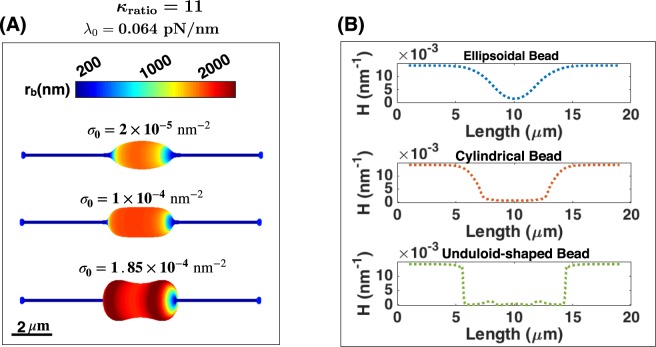
Three different possible shapes of a bead-like structure resulting from the presence of a rigid protein domain. (**A**) Formation of (*i*) an ellipsoidal bead (top) at low protein density, (*ii*) a cylindrical bead (middle) at average protein density, and (*iii*) an unduloid-shaped bead (bottom) at high protein density; ${\kappa }_{{\rm{ratio}}}=11$. The scale bar in panel A is 2 μm. (**B**) The mean curvature (H) distribution along the nanotube length for ellipsoidal (blue line), cylindrical (red), and unduloid-shape (green) beads in panel (A). See Fig. S6 for details of the change in the second derivative of H.

Interestingly, we found that the competition between these two mechanisms leads to the formation of beads with different shapes. Based on the magnitude of the protein density, three different oblate spheroid shapes were obtained – *(i)* an ellipsoidal bead at ${\sigma }_{0}=2\times {10}^{-5}\,{{\rm{nm}}}^{-2}$, *(ii)* a flat cylindrical bead at ${\sigma }_{0}=1\times {10}^{-4}\,{{\rm{nm}}}^{-2}$, and *(iii)* a large unduloid-shaped bead at ${\sigma }_{0}=1.85\times {10}^{-4}{{\rm{nm}}}^{-2}$ (Fig. 5A). These different bead shapes are classified according to the sign of *H*″ (the second derivative of the mean curvature), (*i*) in the ellipsoidal bead, *H*″ is positive everywhere along the bead, (*ii*) in the cylindrical bead, the change in the radius of the bead is very small compared to the radius of bead ($\frac{{\Delta r}_{{\rm{b}}}}{{{\rm{r}}}_{{\rm{b}}}} < 0.01$, we set 0.01 as our threshold), and (*iii*) in the unduloid-shaped bead, *H*″ changes sign along the bead (see Fig. S6).

These results showed that the coupling between two modes of spatial heterogeneity along a membrane nanotube not only increases the radius of a bead (see Fig. S7) but also broadens the energy landscape, enabling the formation of a variety of bead shapes. Furthermore, these shape transitions suggest that the energy landscape of the membrane, which is now modulated by heterogeneities in $\kappa$ and $\sigma$ plays an important role in the shape of the bead.

To further understand the relationship between the mean curvature and the bead shape transition, we plotted the mean curvature distribution that was obtained from our mechanical solution along the nanotube length for three different observed beads shapes in panel A (Fig. 5B). As expected for all shapes, the mean curvature decreases along the beaded domain to lower the energy of the system (Fig. 5B). For the large ellipsoidal bead in panel A, the mean curvature of the middle point of the bead is very small (Fig. 5B, dotted blue line). As the mean curvature becomes very small, the only possible behavior to further decrease the energy of the system is to use the third dimension, arclength ($H(\sigma ,{\kappa }_{{\rm{ratio}}},s)$). Therefore, by increasing the protein density from ${\sigma }_{0}=2\times {10}^{-5}\,{{\rm{nm}}}^{-2}$ to ${\sigma }_{0}=1\times {10}^{-4}\,{{\rm{nm}}}^{-2}$ in Fig. 5A, the mean curvature decreases all along the bead which leads to the formation of the cylindrical bead (Fig. 5B, dotted red line). After the formation of the cylindrical bead, any further increase in the energy of the system causes a buckling instability where the large unduloid-shaped bead forms(Fig. 5B, dotted green line).

### Competition between length scales determines the morphology of the bead-shaped structures along a nanotube

**Figure 6:**
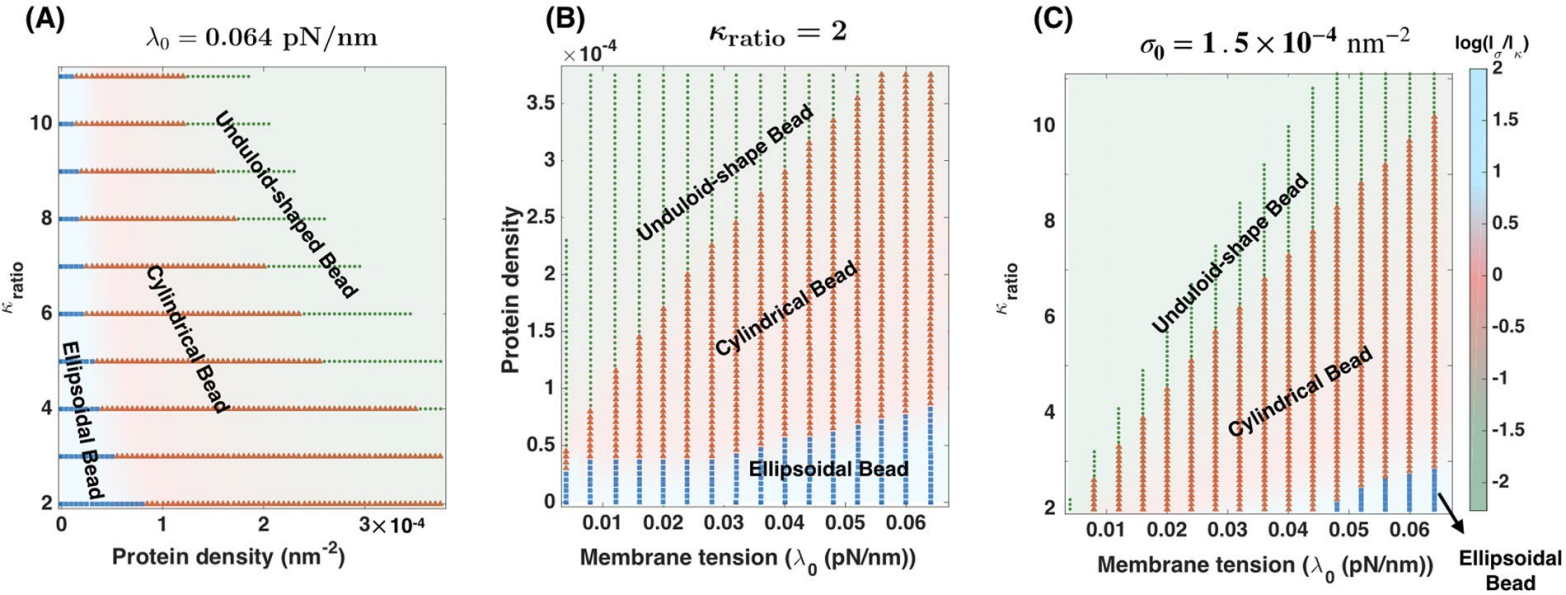
Bead morphology depends on the protein density (*σ*_0_), the bending rigidity ratio of the protein-enriched domain compared to the lipid membrane (${\kappa }_{{\rm{ratio}}}$), and the edge membrane tension *λ*_0_. (**A**) Phase diagram for bending rigidity ratio versus the number of proteins per unit area, ${\lambda }_{0}=0.064\,{\rm{pN}}/{\rm{nm}}$. The background of the phase diagram shows the log of the ratio of the two induced length scales $(\frac{{l}_{\sigma }}{{1}_{\kappa }})$. The three different bead shapes can be distinguished by the dominant length scale: (*i*) ellipsoidal beads when $\log (\frac{{l}_{\sigma }}{{1}_{\kappa }}) > 0$ (blue domain), (*ii*) cylindrical beads when $\log (\frac{{l}_{\sigma }}{{1}_{\kappa }}) \sim 0$ (pink domain), and (*iii*) unduloid-shaped beads when $\log (\frac{{l}_{\sigma }}{{1}_{\kappa }}) < 0$ (green domain). (**B**) The protein density versus the edge membrane tension *λ*_0_ phase diagram for ${\kappa }_{{\rm{ratio}}}\mathrm{=2}$. The background of the phase diagram of the log of the $\frac{{l}_{\sigma }}{{1}_{\kappa }}$ for a range of the membrane tension and the protein density. (**C**) The bending rigidity ratio versus the edge membrane tension ${\lambda }_{0}$ phase diagram for ${\sigma }_{0}=1.5\times {10}^{-4}\,{{\rm{nm}}}^{-2}$. The background of the phase diagram of the log of the $\frac{{l}_{\sigma }}{{1}_{\kappa }}$ for a range of the membrane tension and the bending rigidity ratio. The colors in panels (B,C) represent the same bead shapes as panel (A).

To identify the range of protein density and ${\kappa }_{{\rm{ratio}}}$ over which three different bead shapes in Fig. 5A can form, we performed simulations over a range of protein densities, (${\sigma }_{0}=0-3.75\times {10}^{-4}\,{{\rm{nm}}}^{-2}$), as well as over a range of ${\kappa }_{{\rm{ratio}}}=2-11$, encompassing soft protein domains to very stiff clusters. This variation allowed us to construct a phase diagram to identify the regions of different bead morphologies (Fig. 6A). The pink region represents the formation of ellipsoidal bead-shaped structures, the blue region denotes the cylindrical beads, and the green region indicates the unduloid-shaped beads configuration.

To determine the dominant length scale for each bead shape morphology in Fig. 6A, we compared the two “induced” length scales, one by the rigid domain (${l}_{k}=1/2\sqrt{{\kappa }_{{\rm{protein}}}/\lambda }$) and the other one associated with the protein aggregation (${l}_{\sigma }=1/\mu \varphi \sigma$)^34,42,52^. The background of the phase diagram in Fig. 6A shows that these two length scales act in tandem to regulate the bead size and shape. When ${l}_{\sigma }\gg {l}_{k}$ or $\log (\frac{{l}_{\sigma }}{{l}_{k}}) > 0$, ellipsoidal beads form along the protein-enriched domain (Fig. 6A, blue). When the two length scales become comparable ($\log (\frac{{l}_{\sigma }}{{l}_{k}}) \sim 0$), the formation of cylindrical beads are energetically favorable for the system (Fig. 6A, pink). Finally, at very large values of protein density, when the “induced” length scale by the rigid domain becomes dominant ($\log (\frac{{l}_{\sigma }}{{l}_{k}}) < 0$), the unduloid-shaped bead forms along the membrane nanotube (Fig. 6A, green).

Regardless of which mechanism dominates, the edge tension ${\lambda }_{0}$ implicitly governs the length scale of the membrane; therefore the natural question that arises is: how does this tension govern the shape and length scale transitions of the beads? We explore these questions by conducting two sets of simulations. For the first set of simulations, we varied the edge membrane tension (${\lambda }_{0}$) and the protein density (${\sigma }_{0}$) in a range (${\lambda }_{0}=0.004-0.064\,{\rm{pN}}/{\rm{nm}}$ and ${\sigma }_{0}=0-3.75\times {10}^{-4}\,{{\rm{nm}}}^{-2}$) assuming that ${\kappa }_{{\rm{ratio}}}=2$ (Fig. 6B). We observed that high edge tension shifted the transition of ellipsoidal to cylindrical and unduloid-shaped beads to the large protein densities while the shape transition of the beads is still governed by the ratio of the two induced length scales ${l}_{\sigma }$ and ${l}_{\kappa }$ (Fig. 6B). For the second set of simulations, we fixed the protein density (${\sigma }_{0}=6\times {10}^{-5}\,{{\rm{nm}}}^{-2}$) and varied the edge tension (${\lambda }_{0}$) and the rigidity ratio (${\kappa }_{{\rm{ratio}}}$) between 0.004–0.064 pN/nm and 2–11 respectively (Fig. 6C). As our results show, in this case, all three possible shapes of beads are only formed at high membrane tension and the ratio of the induced length scale govern the morphology of the bead (Fig. 6C). In general, we can see that by increasing the edge membrane tension either at a constant protein density or a fixed rigidity ratio, we decrease the value of the induced length scale by the rigid domain ($\sqrt{{\kappa }_{{\rm{protein}}}/\lambda }$), and therefore we move from the cylindrical and unduloid-shaped beads to the ellipsoidal bead-shaped region of the phase space.

Another aspect of the heterogeneous membrane properties is the variation of the Gaussian modulus between the protein-enriched domain and the bare membrane^90^. Specifically, in the unduloid-shape bead that the Gaussian curvature along the bead changes the sign from positive to negative, the heterogeneity in the Gaussian modulus can play an important role. To explore how the variation in the Gaussian modulus can affect the morphology of the unduloid-shape bead, we repeated the simulation in Fig. 5A (${\sigma }_{0}=1.85\times {10}^{-4}\,{{\rm{nm}}}^{-2}$ and ${\kappa }_{{\rm{ratio}}}=11$) varying the relative Gaussian modulus of the protein-enriched domain between $-20 < \Delta {\kappa }_{G} < 20$ (Fig. S8). As our results show, the variation in the Gaussian modulus has no observable effect on the morphology of the bead and only changes the size of the bead; increasing the Gaussian modulus of the protein-enriched domain respect to the bare membrane decreases the radius of the bead, whereas decreasing it makes the bead larger (Fig. S8).

### Interaction between multiple beads along a nanotube

Often, multiple beads are observed along a membrane nanotube, suggesting that multiple domains of heterogeneity exist along the nanotube^15,17,19,44–47,91^. (Fig. 1A). These observations lead to the following question: how does the profile of these beaded strings depend on the different length scales associated with beaded nanotubes? Previous studies have shown that membrane curvature and tension can control the interaction between two domains of membrane heterogeneities^92–94^. Here, in order to answer this question from the perspective of beading morphology of membrane nanotubes, we conducted simulations for the formation of two beads along the membrane by prescribing two domains of heterogeneity for three cases: (i) varying membrane rigidity alone in each domain in the absence of protein-induced spontaneous curvature, (ii) varying protein density for uniform rigidity, and (iii) varying protein density for domains with higher bending rigidity respect to the bare lipid membrane.

**Figure 7:**
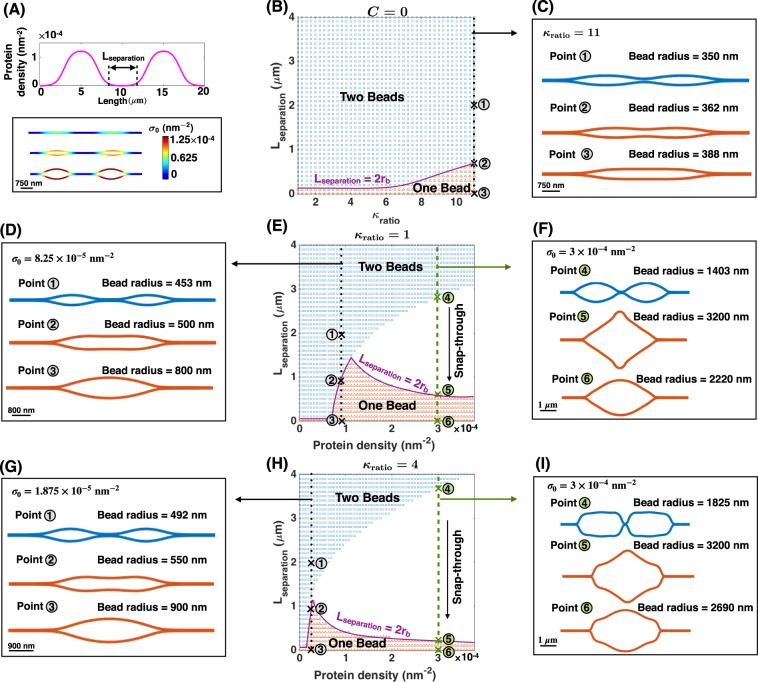
Multiple beads along a nanotube. (**A**, top) Protein density distribution with two domains of protein accumulation. The covered length by each protein-enriched domain is ${{\rm{L}}}_{{\rm{protein}}}=8\,\mu {\rm{m}}$ and the domains are far from each other (${{\rm{L}}}_{{\rm{separation}}}=4\,\mu {\rm{m}}$). The number of proteins within each domain increases by the same amount. (**A**, bottom) Two beads form corresponding to each protein-enriched domain, ${\lambda }_{0}=0.064\,{\rm{pN}}/{\rm{nm}}$. (**B**) The distance between two beads versus the bending rigidity ratio phase diagram with C = 0 and ${\sigma }_{0}\,=\,1.25\times {10}^{-4}\,{{\rm{nm}}}^{-2}$. There are two possible shapes, (*i*) two separated beads denoted with the color blue, and (*ii*) one single bead marked by the color red. The transition from two to one bead is smooth everywhere in this parameter space and occurs when ${{\rm{L}}}_{{\rm{separation}}} < 2{{\rm{r}}}_{{\rm{b}}}$ (purple line). (**C**) Membrane profiles for the marked points along the dotted black line in panel (B). (**D**) Membrane profiles for the black dotted line in panel (E) show the smooth evolution of membrane shape from two beads to one bead at ${\sigma }_{0}=8.25\times {10}^{-5}\,{{\rm{nm}}}^{-2}$ with decreasing ${{\rm{L}}}_{{\rm{separation}}}$ (${\kappa }_{{\rm{ratio}}}=1$). (**E**) Phase diagram for the distance between two beads versus the protein density at ${\kappa }_{{\rm{ratio}}}=1$. The colors represent the same morphologies as panel (B). When ${{\rm{L}}}_{{\rm{separation}}} < 2{{\rm{r}}}_{b}$, there is a smooth transition from two beads to one bead at low protein density and a snap-through instability in the transition from two beads to one bead for large protein densities. (**F**) Membrane profiles show the snap-through transition from two kissing beads to one large bead at ${\sigma }_{0}=3\times {10}^{-4}\,{{\rm{nm}}}^{-2}$ corresponding to the marked points along the green dashed line in panel (E). (**G**) Membrane profiles for the black dotted line in panel (H) show the smooth evolution of membrane shape from two beads to one bead at ${\sigma }_{0}=1.875\times {10}^{-5}\,{{\rm{nm}}}^{-2}$ with decreasing ${{\rm{L}}}_{{\rm{separation}}}$ setting ${\kappa }_{{\rm{ratio}}}=4$. (**H**) Phase diagram for the distance between two beads versus the protein density at ${\kappa }_{{\rm{ratio}}}=4$. The colors represent the same morphologies as panel (B). (**I**) Membrane profiles show the snap-through transition from two kissing beads to one large bead at ${\sigma }_{0}=3\times {10}^{-4}\,{{\rm{nm}}}^{-2}$ for the green dashed line in panel (H).

First, we found when there are two regions of proteins far from each other with an end-to-end distance given by ${{\rm{L}}}_{{\rm{separation}}}=4\,\mu {\rm{m}}$ (Fig. 7A, top), two independent beads form (Fig. 7A bottom). The size and the shape of the beads are independent of the number of domains as long as the domains are far away from each other (Fig. S9). Having established that the regions of heterogeneity are independent when they are far from each other, we next asked under what conditions might these beads interact with one another? In other words, what length scales govern the stability of multiple beads knowing that there is a certain relaxation length between the beads and the cylinder? To answer this question, we repeated the simulation of two beads (Fig. 4), and varying the rigidity ratio (${\kappa }_{{\rm{ratio}}}$) and end-to-end distance (${{\rm{L}}}_{{\rm{separation}}}$) between 1–11 and 0–4 *μ*m respectively (Fig. 7B). Based upon the results, we constructed a phase diagram separating the two possible morphologies; (*i*) two distinct beads represented by the color blue, and (*ii*) one single bead denoted by the color red (Fig. 7B).

We found that when the distance between two rigid domains is shorter than 2 ${{\rm{r}}}_{b}$, there is a smooth transition from two beads connected by a string to a single bead (purple line in Fig. 7B). We observe that the smooth transition in the number of beads is accompanied by the shape transition from an unduloid-shaped bead to a large ellipsoidal bead (Fig. 7C). This suggests that at close distances (${{\rm{L}}}_{{\rm{separation}}} < 2{{\rm{r}}}_{b}$), the energy minimum (Eq. 4) of the nanotube with rigid protein domains is attained for a single large bead rather than for two beads connected by a string. As expected, with increasing ${\kappa }_{{\rm{ratio}}}$, the larger beads form along the nanotube (Fig. 4) and therefore the transition from two beads to one bead occurs even when the beads are far from each other (Fig. 7B). For example, for a very rigid protein domain (${\kappa }_{{\rm{ratio}}}=11$), the transition to one bead happens when ${{\rm{L}}}_{{\rm{separation}}} < 0.8\,\mu {\rm{m}}$ (Fig. 7B). However, for the case that ${\kappa }_{{\rm{ratio}}}=4$, the single bead does not form until the centers of two beads almost overlap (Fig. 7B).

Interestingly, when we varied the separation distance between the beads for a range of protein densities (Fig. 7A), we found a sharp transition from two beads to one bead accompanied by a snap-through instability (Fig. 7D–F). Indeed, for small protein densities, when the separation between the two beads is shorter than 2 ${{\rm{r}}}_{b}$ (purple line in Fig. 7E), the nanotube appears to have one large bead, while the transition from two beads to one bead is continuous indicating that there is no energy barrier or discontinuity to move from one state to another (Fig. 7D and black dotted line in Fig. 7E). However, as the protein density increases, larger beads form along the nanotube and we found the emergence of a snap-through instability for small separation distance (Fig. 7E) corresponding to bead shapes that show a distinct transition from two ellipsoids to a flower-shaped bead to a large ellipsoidal bead (green dashed line in Fig. 7E,F). This means that the landscape between two beads and one bead at high protein densities is governed by a large energy discontinuity, and therefore there is no stable solution for the membrane nanotube in the transition stage from two beads to one bead. The existence of this type of elastic energy discontinuity is also observed between two neighboring embedded nanoparticles in membranes^95–97^.

Finally, when we repeated these calculations for a rigid protein domain such as ${\kappa }_{{\rm{ratio}}}=4$ (Fig. 7G–I), we found that the distance and protein density still govern the energy and stability landscape, but the transition point, where the snap-through instability occurs, is shifted towards the lower protein densities, with no change in bead shapes (Fig. 7G–I). This is because for rigid protein domains, larger beads form along the nanotube compared to the uniform bending rigidity in Fig. 7D,G. Therefore, the snap-through transition from two beads to one bead for the rigid protein domain occurs in smaller values of the protein density (Fig. 7H). Generally, our results in Fig. 7 indicate that individual beads along nanotubes potentially tend to be dominant because any fusion of two beads requires overcoming an energy discontinuity with non-trivial shape transitions.

## Discussion

Tunneling nanotubes are membranous projections between cells^1,2,17^. Much of the biophysics associated with these dynamic structures are only beginning to be explored but it is becoming increasingly clear that the cellular membrane and membrane-protein interactions play a critical role in maintaining these cellular architectures^15,19,73,86,98^. In this study, we explored how the energy landscape and the role of heterogeneity in the membrane either due to protein aggregation or material properties alter the architecture of nanotubes. Our results can be summarized as follows – membrane heterogeneity due to either protein-induced spontaneous curvature (Fig. 3) or membrane rigidity (Fig. 4) can result in the formation of bead-like structures along a nanotube. Additionally, the interaction between these two modes of heterogeneity can lead to the formation of beads with distinct shapes while the transitions between these shapes from ellipsoidal to cylindrical to unduloid-shaped beads are consequences of energy minimization and competing length scales in the system (Figs. 5 and 6). Finally, we found that there is an energy discontinuity that impedes any fusion of two beads which suggests the formation of multiple stable beads along the nanotube due to membrane heterogeneity (Fig. 7).

Interaction between membrane inclusions has been studied extensively^99–102^. The membrane inclusions may attract or repel each other depending on the local membrane deformation due to the induced spontaneous curvature or the hydrophobic mismatch of the membrane inclusion^100,103^. For instance, Gil *et al*. showed that the interaction between two adjacent inclusions is attractive if both inclusions change the membrane thickness in the same manner (decrease or increase membrane thickness)^104^. Phillips *et al*.^105^ and later Simunovic *et al*.^106^ demonstrated that there is an attractive force between inclusions with opposite intrinsic curvature. Even in the case of the nano particles, it is suggested that the long-range Casimir-like forces in the fluctuating membrane can induce attractive forces between two neighboring particles^107^. Despite the rich literature on the interaction of the membrane inclusions, the shape of membrane nanotubes resulting from the interplay between a spatially varying membrane rigidity and protein-induced spontaneous curvature has not been previously explored. Our findings should be a motivation for future studies to investigate the interaction between two regions of heterogeneity, particularly for estimating the effective force between two beads and how membrane tension, lipid flow, and other force generating mechanisms can regulate this force between two domains of heterogeneity.

Various recent studies have demonstrated that inducing a constant homogeneous membrane tension along a cylindrical membrane can also lead to a dramatic shape transformation into a modulated structure of a string of pearls^25–30^. However, the tension of lipid membranes can change not only globally but also locally due to the absorption of proteins, nanoparticles, inclusions, or actomyosin interactions with the membrane^34–38^. Here, we show that local variation of the membrane tension corresponding to the membrane heterogeneities in the beaded nanotubes (Figs. S3 and S4) may play a role in governing the morphology of the membrane nanotubes. In addition to the membrane nanotubes, the beaded morphologies have been observed on different membrane structures which make direct connect with the extracellular matrix^108,109^. The membrane mechanics of these “beaded apotopodia” is still a matter of the debate^110^. However, we anticipate that an extension of our membrane mechanical model can be a powerful tool to understand the physics behind the formation of these unusual beaded structures.

Our simulations lead to the following predictions. Tension at the edge of the nanotube not only governs the nanotube radius but also its response to heterogeneity. Therefore, manipulating cell tension and evaluating how it affects the morphology of the nanotube will provide information on how the tensional homeostasis of cells affects the membrane nanotubes. Additionally, we found that there is an energy discontinuity that governs the landscape of the transition from two beads to one bead. This energy discontinuity, governed by a snap-through instability, suggests that the fusion of two beads depends on the membrane composition and its material properties such that under high protein density or high rigidity conditions, there is a large crossover energy discontinuity for fusion. These predictions can be extended to multiple beads as well.

In the broader context of interactions between the bilayer membrane and curvature inducing moieties (proteins and cytoskeletal filaments), Shlomovitz and Gov showed that the coupling between membrane shapes and membrane-bound FtsZ filaments can induce high-density FtsZ rings along a cylindrical membrane^91^. With no entropic effects, these rings interact with each other, can coalesce and form larger rings depending on the membrane tension and separation distances. They predict that when the separation between two rings is larger than 2 *π*R (R is the radius of the cylinder), membrane shape undulations around each ring act as an energy barrier to stabilize the separate rings and preventing coalescence^91^. These results suggest that the observed energy discontinuity in our model and Shlomovitz and Gov paper could have a similar origin since both responses appear due to the elastic behavior of the lipid membranes in interactions with local curvature inducing moieties.

Recent experiments on the fission of yeast have demonstrated that the formed rings along the tubular membranes by the actin-myosin contractile force interact and fuse when the natural width of the ring is much smaller than their separation distance^91,111^. This is also consistent with our simulation results, as we found that the transition from two beads to one bead occurs when the distance between two beads is shorter than the diameter of the beads (${{\rm{L}}}_{{\rm{separation}}} < 2{{\rm{r}}}_{b}$) (Fig. 7). Ultimately, to explore the instability that we observed in the interaction of two beads, futures studies will be needed to focus on detailed non-linear stability analysis for large deformations including tension and shape coupling and without the restriction of small deformations and linearization^78,91^.

Although our model has provided several fundamental insights into the role of membrane composition in nanotube morphology, we have made simplifying assumptions that may need to be revisited as more experimental evidence is gathered regarding these structures. Additionally, the dynamics of membrane-protein diffusion and what could be the underlying mechanisms that govern protein aggregation along the nanotube as suggested in our model are not yet fully explored. While there is evidence for the strong curvature-mediated feedback for the protein aggregation^64^, it is possible that feedback between proteins in the lumen of the nanotube and biochemical signaling can lead to the formation of protein microdomains. For example, it is known that phase separation between two main components of the membrane – clusters of sphingolipids and cholesterol molecules – can result in the formation of lipid rafts^89,112^.

In addition, the role of the cytoskeleton (actin and microtubule filaments) and motor protein transport along the nanotube is known to be an important contributor^1,2,113^, but a correlation with the beading morphology is yet to be established. Also, there are only a handful of direct experimental observations of membrane heterogeneities along nanotubes in cells^44^. And finally, the role of the active transport versus the flow of cytosolic components in governing the stability of these nanotubes is not captured by our model and remains an active focus of our research and modeling efforts.

## Supplementary information

Supplementary material
