# Supplementary material for: Modeling membrane nanotube morphology: the role of heterogeneity in composition and material properties
Source: Sci Rep. 2020 Feb 13;10:2527. doi: 10.1038/s41598-020-59221-x (PMC7018976; doi:10.1038/s41598-020-59221-x)
Supplement: Supplementary file 1 — Supplementary material [file 41598_2020_59221_MOESM1_ESM.pdf]

Supplementary material for "Modeling membrane nanotube morphology: the role of heterogeneity in composition and material properties"

H. Alimohamadi, B. Ovryn, and P. Rangamani*

January 21, 2020

Contents

1 Methods **2**

1.1 Membrane mechanics . . . 2

1.2 Helfrich energy and mechanical equilibrium . . . 3

1.3 Governing equations in axisymmetric coordinates . . . 4

1.4 Analytical solutions (limit cases) . . . 6

1.5 Numerical implementation . . . 7

2 Tables **8**

2.1 Table of Notation . . . 8

2.2 Table of parameters . . . 10

3 Supplementary Figures **11**

*prangamani@ucsd.edu

1 Methods

1.1 Membrane mechanics

In this section, we present a brief derivation of the generalized equations of motion for biological membranes under the assumptions stated in the main text. We then restrict the equations to axisymmetric coordinates and solve them in a prescribed membrane domain. Details of all the derivations can be found in [1–3].
The local force balance in the absence of inertia for any material point on the membrane is given by

$$\boldsymbol{\Gamma}^{\xi}_{;\xi} + p\mathbf{n} = 0, \tag{S1}$$

where $()_{;\xi}$ is the surface divergence, $\boldsymbol{\Gamma}^{\xi}$ is the stress vector, p is the pressure difference between the inside and outside of the volume bounded by the membrane, and $\mathbf{n}$ is the unit vector normal to the membrane. The surface divergence in Eq. (S1) can be rewritten as [1]

$$\boldsymbol{\Gamma}^{\xi}_{;\xi} = (\sqrt{a})^{-1}(\sqrt{a}\boldsymbol{\Gamma}^{\xi})_{,\xi}, \tag{S2}$$

where a is the determinant of the first fundamental form metric $a_{\xi\eta}$ and $()_{,}$ is the partial derivative with respect to the coordinate. The stress vector in Eq. (S1) can be decomposed into normal and tangential components as

$$\boldsymbol{\Gamma}^{\xi} = \boldsymbol{T}^{\xi} + S^{\xi}\mathbf{n}. \tag{S3}$$

For elastic surfaces for which the energy density per unit mass $F(a_{\xi\eta}, b_{\xi\eta})$ depends on the first and second fundamental forms, the normal and tangential components of the stress vector in Eq. (S3) can be expressed as [1, 4]

$$\mathbf{T}^{\xi} = T^{\xi\eta}\mathbf{a}_{\eta} \quad \text{with} \quad T^{\xi\eta} = \Gamma^{\xi\eta} + b^{\eta}_{\mu}M^{\mu\xi}, \quad \text{and} \quad S^{\xi} = -M^{\xi\eta}_{;\eta}, \tag{S4}$$

where

$$\Gamma^{\xi\eta} = \rho\left(\frac{\partial F(\rho,H,K;x^{\xi})}{\partial a_{\xi\eta}} + \frac{\partial F(\rho,H,K;x^{\xi})}{\partial a_{\eta\xi}}\right), \quad M^{\xi\eta} = \frac{\rho}{2}\left(\frac{\partial F(\rho,H,K;x^{\xi})}{\partial b_{\xi\eta}} + \frac{\partial F(\rho,H,K;x^{\xi})}{\partial b_{\eta\xi}}\right). \tag{S5}$$

Here, ρ is the surface mass density, and H and K are the mean and Gaussian curvatures given by

$$H = \frac{1}{2}a^{\xi\eta}b_{\xi\eta}, \quad K = \frac{1}{2}\varepsilon^{\xi\eta}\varepsilon^{\lambda\mu}b_{\xi\lambda}b_{\eta\mu}. \tag{S6}$$

Here $(a^{\xi\eta}) = (a_{\xi\eta})$ is the dual metric and $\varepsilon^{\xi\eta}$ is the permutation tensor defined by $\varepsilon^{12} = -\varepsilon^{21} = 1/\sqrt{a}, \varepsilon^{11} = \varepsilon^{22} = 0$.

In the case of area incompressibility ($J = 1$), the general form of free energy density per unit mass can be rewritten as

$$F(\rho,H,K;x^{\xi}) = \tilde{F}(H,K;x^{\xi}) - \frac{\gamma(x^{\xi},t)}{\rho}, \tag{S7}$$

where $\gamma(x^{\xi},t)$ is a Lagrange multiplier field required to impose invariance of ρ on the whole of the surface (see [1] for full derivation). Substituting $W = \rho\tilde{F}$ into Eq. (S7) we get

$$\Gamma^{\xi\eta} = (\lambda + W)a^{\xi\eta} - (2HW_H + 2\kappa W_K)a^{\xi\eta} + W_H\tilde{b}^{\xi\eta}, \tag{S8}$$

$$M^{\xi\eta} = \frac{1}{2}W_H a^{\xi\eta} + W_K\tilde{b}^{\xi\eta}, \tag{S9}$$

where $\tilde{b^{\xi\eta}}$ is the co-factor of the curvature tensor, and $\lambda = -(\gamma + W)$ can be interpreted as the membrane tension [5, 6].

Combining Eqs. (S9), (S4), and (S3) with Eq. (S1) gives the equation of motion in normal and tangential directions as

$$\text{Normal:} \quad p = \Delta\frac{1}{2}W_H + (W_K)_{;\xi\eta}\tilde{b}^{\xi\eta} + W_H(2H^2 - K) + 2H(KW_K - W) - 2\lambda H, \tag{S10}$$

and

$$\text{Tangential:} \quad N^{\eta\xi}_{;\xi} - S^{\xi}b^{\eta}_{\xi} = -(\gamma_{,\xi} + W_K k_{,\xi} + W_H H_{,\xi})a^{\eta\xi} = (\frac{\partial W}{\partial x^{\xi}}|_{\text{exp}} + \lambda_{,\xi})a^{\eta\xi} = 0. \tag{S11}$$

Here $\Delta(\cdot)$ is the surface Laplacian, and $()_{|\text{exp}}$ denotes the explicit derivative respect to coordinate θ^{ξ}.

1.2 Helfrich energy and mechanical equilibrium

For the local energy density of a lipid bilayer membrane, we use an augmented version of the Helfrich energy accounting for the protein-protein interaction, the protein density gradient and the thermal entropic contributions given by [7–11]

$$W(H,K,\sigma;\theta^{\xi}) = \underbrace{\kappa(\theta^{\xi})\left[H - C(\sigma(\theta^{\xi}))\right]^2 + \kappa_G(\theta^{\xi})K}_{\text{Bending}} \underbrace{-\alpha\sigma(\theta^{\xi})^2}_{\text{Protein aggregation}} + \underbrace{\beta(\nabla\sigma)^2}_{\substack{\text{Inhomogeneous}\\ \text{protein distribution}}} + \underbrace{k_BT\sigma(\log(\frac{\sigma}{\sigma_s}) - 1)}_{\substack{\text{Entropic contribution}\\ \text{due to thermal diffusion}}}, \tag{S12}$$

where W is the local energy density, C is the induced spontaneous curvature due to lipid-protein interactions, σ is the protein density, κ is the bending modulus, κ_G is the Gaussian modulus, α indicates the strength of the attractive energy between two neighboring proteins [7, 10], β is a positive constant that depends on the excluded area and the effective interaction area of the proteins [12, 13], ∇ is the gradient operator, k_B is the Boltzmann constant, and T is the temperature. Assuming that the system in the room temperature ($k_BT = 4.114$ pN $\cdot$ nm), we note that in Eq. S12, $\frac{k_BT}{\kappa}$ is small because the membrane bending rigidity is in the range of 20-40 k_BT [14]. Additionally, in the dilute regime of low protein density $k_BT\sigma \ll 1$. Based on this analysis, we neglect the entropic term in the rest of our calculations.

In Eq. (S12), protein density (σ) depends explicitly on the surface coordinates θ^{ξ} to allow for local heterogeneity. It should be noted that the bending term in Eq. (S12) is different from the standard Helfrich energy by a factor of 2. We take this net effect into consideration by choosing the value of the bending modulus to be twice that of the standard value of bending modulus typically used for lipid bilayers [15].

For low protein densities (dilute regime), the induced-spontaneous curvature $C(\sigma)$ in Eq. (S12) can be expressed in term of protein density as [16]

$$C(\sigma) = (\mu\varphi)\sigma, \tag{S13}$$

where φ is the angle between cone-shaped proteins meridian and the normal vector to the surface ($\mathbf{n}$), and μ is a length-scale correction representing the lipid-protein specific interaction [17].

Substituting the modified version of Helfrich energy function (Eq. (S12)) and Eq. (S13) into the first functional normal variation of total energy (Eq. (S10)) gives the so-called "shape equation," [18]

$$\underbrace{\Delta\left[\kappa\left(H-(\mu\varphi)\sigma\right)\right]-(\kappa_G)_{;\xi\eta}\tilde{b}^{\xi\eta}-2\kappa H\left(H-(\mu\varphi)\sigma\right)^2+2\kappa\left(H-(\mu\varphi)\sigma\right)\left(2H^2-K\right)}_{\text{Elastic effects}}$$
$$+\underbrace{2H(\alpha\sigma^2)}_{\text{Protein aggregation}}-\underbrace{2H\beta(\nabla\sigma)^2)}_{\substack{\text{Inhomogeneous}\\ \text{protein distribution}}}=\underbrace{(p+2\lambda H)}_{\substack{\text{Capillary}\\ \text{effect}}}, \tag{S14}$$

A consequence of the spatial variation of membrane properties and protein density is that λ is not homogeneous along the membrane [5, 19, 20]. Substituting Eq. (S12) into the balance of forces tangential to the membrane Eq. (S11) gives the spatial variation of membrane tension,

$$\underbrace{\nabla\lambda}_{\substack{\text{Gradient of}\\ \text{membrane tension}}}=\underbrace{2[\kappa\mu\varphi(H-(\mu\varphi)\sigma)+\alpha]\frac{\partial\sigma}{\partial\theta^\xi}-\beta(\nabla\sigma)\frac{\partial(\nabla\sigma)}{\partial\theta^\xi}}_{\text{Protein density variation}}-\underbrace{\frac{\partial\kappa}{\partial\theta^\xi}(H-(\mu\varphi)\sigma)^2}_{\substack{\text{Bending modulus-}\\ \text{induced variation}}}-\underbrace{\frac{\partial\kappa_G}{\partial\theta^\xi}K}_{\substack{\text{Gaussian modulus-}\\ \text{induced variation}}}. \tag{S15}$$

1.3 Governing equations in axisymmetric coordinates

We define a surface of revolution (Fig. 1C) by

$$\mathbf{r}(s,\theta)=R(s)\mathbf{e}_r(\theta)+Z(s)\mathbf{k}, \tag{S16}$$

where s is the arc length along the curve, R(s) is the radius from the axis of rotation, Z(s) is the height from the base plane and $(\mathbf{e}_r,\mathbf{e}_\theta,\mathbf{k})$ form the basis coordinate. Defining ψ as the angle made by the tangent with respect to the vertical gives

$$R'(s)=\cos(\psi),\quad Z'(s)=\sin(\psi), \tag{S17}$$

satisfying the identity $(R')^2+(Z')^2=1$, where $(')$ is the partial derivative with respect to the arc length. Using this, we can define the normal and tangent vectors to the surface as

$$\mathbf{n}=-\sin\psi\mathbf{e}_r(\theta)+\cos\psi\mathbf{k},\quad \mathbf{a}_s=\cos\psi\mathbf{e}_r(\theta)+\sin\psi\mathbf{k}. \tag{S18}$$

Using this parameterization, we can now write the tangential (κ_ν) and transverse (κ_τ) curvatures as

$$\kappa_\nu=\psi',\quad \kappa_\tau=\frac{\sin\psi}{R}. \tag{S19}$$

The mean curvature (H) and Gaussian curvature (K) are obtained by summation and multiplication of the tangential and transverse curvatures

$$H=\frac{1}{2}(\kappa_\nu+\kappa_\tau)=\frac{1}{2}(\psi'+\frac{\sin\psi}{R}),\quad K=\kappa_\tau\kappa_\nu=\frac{\psi'\sin\psi}{R}. \tag{S20}$$

Finally, we define $L=\frac{1}{2\kappa}R(W_H)'$ such that we obtain a system of first- order differential equations with six unknowns R, Z, ψ, H, L, and λ [18, 20, 21],

$$
\begin{aligned}
R' = \cos\psi, \quad Z' = \sin\psi, \quad R\psi' = 2RH - \sin\psi, \quad RH' = L + R\mu\varphi\sigma' - \frac{R\kappa'}{\kappa}(H - \mu\varphi\sigma), \\
\frac{L'}{R} = \frac{p}{k} + 2H\left[(H - (\mu\varphi)\sigma)^2 - \frac{\alpha\sigma^2}{\kappa} + \frac{\beta(\nabla\sigma)^2}{\kappa} + \frac{\lambda}{\kappa}\right] \\
-2\,(H - (\mu\varphi)\sigma)\left[H^2 + \left(H - \frac{\sin\psi}{R}\right)^2\right] - \frac{\kappa' L}{\kappa R} - \frac{\kappa_G'' \sin(\psi)}{\kappa R} - \frac{\kappa_G' \cos(\psi)}{\kappa R}(2H - \frac{\sin(\psi)}{R}), \\
\lambda' = 2\Big[\kappa\mu\varphi\,(H - \mu\varphi\sigma) + \alpha\sigma - \beta\sigma''\Big]\sigma' - \kappa'(H - (\mu\varphi)\sigma)^2 - \kappa_G' K.
\end{aligned}
\tag{S21}
$$

In order to solve this system of equations (Eq. (S21)), we need to provide six boundary conditions. We consider an axisymmetric cylindrical membrane with (1) fixed radius at both boundaries, (2) $\psi = \pi/2$ at both ends to ensure the edges remain vertical, (3) fixed height at one end and prescribed tension (λ_0) at the other end (see Fig. 1C). These boundary conditions can be summarized as follow,

$$
\begin{aligned}
R(0^+) = R_0, \quad \psi(0^+) = \frac{\pi}{2}, \quad z(0^+) = 0 \quad \lambda(0^+) = \lambda_0, \\
R(s_{\text{max}}) = R_0, \quad \psi(s_{\text{max}}) = \frac{\pi}{2}.
\end{aligned}
\tag{S22}
$$

To non-dimensionalize the system of equations (Eq. (S21)), we use two parameters, the radius of the initial cylindrical membrane (R_0), and lipid bilayer bending modulus (κ_0). Using these constants, we can define

$$
\begin{aligned}
t = \frac{s}{R_0}, \quad y = \frac{Z}{R_0}, \quad x = \frac{R}{R_0}, \quad h = HR_0, \quad c = CR_0, \quad l = LR_0, \quad \tilde{\lambda} = \frac{\lambda R_0^2}{\kappa}, \quad G = KR_0^2 \\
\tilde{p} = \frac{pR_0^3}{\kappa_0}, \quad \tilde{\kappa}_G = \frac{\kappa_G}{\kappa}, \quad \tilde{\kappa} = \frac{\kappa}{\kappa_0}, \quad \tilde{\alpha} = \frac{\alpha}{R_0^2\kappa_0}, \quad \tilde{\beta} = \frac{\beta}{R_0^4\kappa_0}, \quad \tilde{\mu} = \frac{\mu}{R_0}, \quad \tilde{\sigma} = \frac{\sigma}{R_0^2}.
\end{aligned}
\tag{S23}
$$

Rewriting Eq. (S21) in terms of the dimensionless variables in Eq. (S23), we get [20]

$$
\begin{aligned}
\dot{x} = \cos\psi, \quad \dot{y} = \sin\psi, \quad x\dot{\psi} = 2xh - \sin\psi, \quad x\dot{h} = l + x\tilde{\mu}\varphi\dot{\tilde{\sigma}} - x\frac{\dot{\tilde{\kappa}}}{\tilde{\kappa}}(h - \tilde{\mu}\varphi\tilde{\sigma}), \\
x^{-1}\dot{l} = \frac{\tilde{p}}{\tilde{\kappa}} + 2h\left[(h - (\tilde{\mu}\varphi)\tilde{\sigma})^2 - \frac{\tilde{\alpha}\tilde{\sigma}^2}{\tilde{\kappa}} + \frac{\tilde{\beta}(\nabla\tilde{\sigma})^2}{\tilde{\kappa}} + \frac{\tilde{\lambda}}{\tilde{\kappa}}\right] - 2\,(h - (\tilde{\mu}\varphi)\tilde{\sigma})\left[h^2 + \left(h - \frac{\sin\psi}{x}\right)^2\right] - \frac{\dot{\tilde{\kappa}}}{\tilde{\kappa}}x^{-1}l - \\
x^{-1}\frac{\ddot{\tilde{\kappa}}_G}{\tilde{\kappa}}\sin(\psi) - x^{-1}\frac{\dot{\tilde{\kappa}}_G}{\tilde{\kappa}}\cos(\psi)(2h - \frac{\sin(\psi)}{x}), \\
\dot{\tilde{\lambda}} = 2\Big[\tilde{\kappa}\tilde{\mu}\varphi\,(H - \tilde{\mu}\varphi\tilde{\sigma}) + \tilde{\alpha}\tilde{\sigma} - \tilde{\beta}\ddot{\tilde{\sigma}}]\dot{\sigma} - \dot{\tilde{\kappa}}(h - (\tilde{\mu}\varphi)\tilde{\sigma})^2 - \dot{\tilde{\kappa}}_G G,
\end{aligned}
\tag{S24}
$$

where $\dot{()}$ is the derivative respect to the non-dimensional variable t. The boundary conditions in Eq. (S22) simplify to

$$
\begin{aligned}
x(0^+) = 1, \quad \psi(0^+) = \frac{\pi}{2}, \quad y(0^+) = 0, \quad \tilde{\lambda}(0^+) = \tilde{\lambda}_0 \\
x(t_{\text{max}}) = 1, \quad \psi(t_{\text{max}}) = \frac{\pi}{2},
\end{aligned}
\tag{S25}
$$

where $t_{\text{max}} = s_{\text{max}}/R_0$ is the total dimensionless membrane length .

1.4 Analytical solutions (limit cases)

Ignoring the boundary effects and Gaussian curvature, for a nanotube with a length of L_c, radius R_c, uniform bending rigidity and no protein distribution ($\sigma = 0$), the free energy density (Eq. S12) can be written

$$W_0 = \kappa H_0^2 = \kappa(\frac{1}{2R_c}^2), \tag{S26}$$

where H_0 is the initial mean curvature that for a cylinder is proportional to the radius ($H_0 = \frac{1}{2R_c}$). Adding the proteins or having a heterogenous bending rigidity changes the energy of the system as

$$W = \kappa H^2 + \kappa(\mu\varphi\sigma)^2 - 2\kappa\mu\varphi\sigma - \alpha\sigma^2 + \beta(\nabla\sigma)^2, \tag{S27}$$

where we assumed that neither topology nor boundary change and eliminated the Gaussian curvature term from the energy. Rearranging the terms in Eq. S27, gives

$$W = \kappa H^2 - 2\kappa\mu\varphi\sigma + \sigma^2(\kappa(\mu\varphi)^2 - \alpha) + \beta(\nabla\sigma)^2. \tag{S28}$$

Since $\varphi < 0$, the second term in Eq. (S28) is positive. Also, for the set of parameters that we choose in this study, the third term in Eq. (S28) is positive. Therefore, we can conclude that adding protein density or varying the bending rigidity increases the energy of the system ($W \geq W_0$). However, any system wants to have minimum energy. Thus, the mean curvature in Eq. (S27) varies in order to decrease the energy of the system. Considering the limit case that $W = W_0$, we find an analytical expression for the mean curvature as a function of the protein density and the bending rigidity as

$$H_{\text{analytical}} = \underbrace{\mu\varphi\sigma}_{\substack{\text{Spontaneous}\\ \text{curvature}}} \pm \sqrt{\underbrace{\frac{\frac{1}{(2R_c)^2}}{\kappa_{\text{ratio}}}}_{\substack{\text{Preexisting curvature}\\ \text{of the tube}}} + \underbrace{\frac{\frac{\alpha\sigma^2}{\kappa}}{\kappa_{\text{ratio}}}}_{\substack{\text{Aggregation}\\ \text{effects}}} - \underbrace{\frac{\frac{\beta(\nabla\sigma)^2}{\kappa}}{\kappa_{\text{ratio}}}}_{\substack{\text{Inhomogeneous}\\ \text{protein distribution}}}}, \tag{S29}$$

where κ_{ratio} represents the ratio of the bending rigidity in the protein-enriched domain (κ_{rigid}) compared to the bending rigidity of the bare lipid membrane ($\kappa_{\text{ratio}} = \kappa_{\text{protein}}/\kappa$). In Eq. (S29), the positive sign is only acceptable because the first term is negative ($\varphi < 0$), and therefore to have a positive curvature along the nanotube, the second term needs to be positive.

For small protein density ($\sigma \ll 1$), the higher order terms in Eq. 7equation.0.7 can be ignored and the equation can be simplified by the Taylor expansion given by

$$H_{\text{analytical}} = \underbrace{\mu\varphi\sigma}_{\substack{\text{Spontaneous}\\ \text{curvature}}} + \frac{1}{\sqrt{\kappa_{\text{ratio}}}}\Big(\underbrace{\frac{1}{(2R_c)}}_{\substack{\text{Preexisting curvature}\\ \text{of the tube}}} + \frac{R_c}{\kappa}(\underbrace{\alpha\sigma^2}_{\substack{\text{Aggregation}\\ \text{effects}}} - \underbrace{\beta(\nabla\sigma)^2}_{\substack{\text{Inhomogeneous}\\ \text{protein distribution}}} \Big), \tag{S30}$$

Assuming that the nanotube remains as a cylinder after deformation ($H_{\text{analytical}} = \frac{1}{2r_{\text{b, analytical}}}$), where $r_{\text{b,analytical}}$ is the radius of the beaded nanotube, Eq. (S29) simplifies as

$$2r_{\text{b,analytical}} = \left[\mu\varphi\sigma + \sqrt{\frac{\frac{1}{(2R_c)^2} + \frac{\alpha\sigma^2}{\kappa} - \frac{\beta(\nabla\sigma)^2}{\kappa}}{\kappa_{\text{ratio}}}} \right]^{-1}. \tag{S31}$$

Similar to Eq. S30, we can simplify Eq. S31 for small protein density given by

$$r_{\text{b,analytical}} = \sqrt{\kappa_{\text{ratio}}} R_{\text{c}} \Big(1 - 2R_{\text{c}}\sqrt{\kappa_{\text{ratio}}}(\mu\varphi\sigma) - \frac{2R_{\text{c}}^2}{\kappa}(\alpha\sigma^2 - \beta(\nabla\sigma)^2)\Big). \tag{S32}$$

1.5 Numerical implementation

We solved the system of first-order differential equations (Eq. (S24)) with boundary conditions Eq. (S25) using the finite element software COMSOL MULTIPHYSICS ® 5.3, using the "General Form PDE" module. Here, we summarize the steps and assumptions that we used for each simulation.

- All the simulations were performed for a fixed length of the membrane ($L_{\text{membrane}} = 20\ \mu\text{m}$).
- The membrane patch was initialized to be a perfect cylinder ($\psi = \pi/2$) with radius R_0= 200 nm.
- The membrane domain (0-s_{max}) was discretized equally with mesh size = 0.001.
- To have a sharp but smooth transition in heterogeneous properties, we used a hyperbolic tangent function given by

$$\chi = \frac{1}{2}[\tanh(g(t - s_0))], \tag{S33}$$

  where χ denoted the membrane property such as bending modulus (κ), Gaussian modulus (κ_G), or protein density (σ), g is the length of the transition regions between the protein-enriched domain and the bare membrane [13], and s_0 represents the domain of the protein aggregation.
- The applied tension at the boundary (λ_0) in Fig. S1, the number of proteins per unit area (σ_0) in Fig.3, and the bending modulus ratio (κ_{ratio}) in Fig. 4 were progressively increased such that each solution was used as an initial guess for the next step.

2 Tables

2.1 Table of Notation

Table 1: Notation used in the model

Notation	Description	Units
E	Strain energy	pN · nm
γ	Lagrange multiplier for incompressibility constraint	pN$\cdot\mathrm{nm}^{-1}$
p	Pressure difference across the membrane	pN$\cdot\mathrm{nm}^{-2}$
σ	Protein density	nm^{-2}
C	Spontaneous curvature	nm^{-1}
θ^{ξ}	The surface coordinatwe	
W	Local energy per unit area	pN$\cdot\mathrm{nm}^{-1}$
$\mathbf{r}$	Position vector	
$\mathbf{n}$	Normal to the membrane surface	unit vector
$\boldsymbol{\nu}$	Tangent to the membrane surface in direction of increasing arc length	unit vector
$\boldsymbol{\tau}$	Rightward normal in direction of revolution	unit vector
$\mathbf{a}_{\xi}$	Basis vectors describing the tangent plane	
λ	Membrane tension, $-(W+\gamma)$	pN/nm
H	Mean curvature of the membrane	nm^{-1}
K	Gaussian curvature of the membrane	nm^{-2}
κ_{ν}	Tangential curvature	nm^{-1}
κ_{τ}	Transverse curvature	nm^{-1}
κ	Bending modulus	pN · nm
$k_B T$	Boltzman energy	pN · nm
κ_G	Gaussian modulus	pN · nm
s	Arclength	nm
θ	Azimuthal angle	
ψ	Angle between $\mathbf{e}_r$ and $\mathbf{a}_s$	
φ	Angle between normal vector to surface $\mathbf{n}$ and protein meridian	
α	Protein-Protein aggregation strength	nm$^3\cdot$ pN
β	Constant representing the excluded area in membrane-protein interaction	nm$^5\cdot$ pN
μ	Constant indicating lipid-protein moietic length scale	nm
R	Radial distance	nm
Z	Elevation from base plane	nm
$\mathbf{e}_r(\theta)$	Radial basis vector	unit vector
$\mathbf{e}_{\theta}$	Azimuthal basis vector	unit vector
$\mathbf{k}$	Altitudinal basis vector	unit vector
s_{max}	Maximum arclength	nm

Table 2: Notation used in the model

Notation	Description	Units
λ_0	Surface tension at boundary	pN$\cdot \mathrm{nm}^{-1}$
σ_0	Number of proteins per unit area	nm^{-2}
$\mathrm{L_c}$	Length of cylinder	nm
$\mathrm{R_c}$	Radius of cylinder	nm
r_b	Radius of bead	nm
L	Shape equation variable	nm^{-1}
γ	Wave number	nm^{-1}
x	Dimensionless radial distance	
y	Dimensionless height	
h	Dimensionless mean curvature	
c	Dimensionless spontaneous curvature	
l	Dimensionless L	
$\tilde{\lambda}$	Dimensionless surface tension	
$\tilde{\lambda_0}$	Dimensionless surface tension at the boundary	
$\tilde{p}$	Dimensionless pressure	
$\tilde{\kappa}$	Dimensionless bending modulus	
$\tilde{\kappa}_G$	Dimensionless Gaussian modulus	
$\tilde{\sigma}$	Dimensionless protein density	
$\tilde{\mu}$	Dimensionless constant μ	
$\tilde{\alpha}$	Dimensionless constant α	
$\tilde{\beta}$	Dimensionless constant β	
G	Dimensionless Gaussian curvature	
$\mathrm{L_{separation}}$	Distance between two beads	μm
$\mathrm{L_{rigid}}$	Length of the rigid segment	μm
$\mathrm{L_{protein}}$	Length of the coat protein	μm
κ_{lipid}	Bending modulus of the bare lipid bilayer	$\mathrm{pN} \cdot \mathrm{nm}$
$\kappa_{\mathrm{protein}}$	Bending modulus of the rigid protein domain	$\mathrm{pN} \cdot \mathrm{nm}$
κ_{ratio}	Bending rigidity ratio $\kappa_{\mathrm{protein}}/\kappa$	
$\kappa_{\mathrm{G,lipid}}$	Gaussian modulus of the bare lipid bilayer	$\mathrm{pN} \cdot \mathrm{nm}$
$\kappa_{\mathrm{G,protein}}$	Gaussian modulus of the protein domain	$\mathrm{pN} \cdot \mathrm{nm}$
$H_{\mathrm{analytical}}$	Aanalytical mean curvature along the protein enrich-domain derived for a special case	nm^{-1}
$\mathrm{r_{b,analytical}}$	Aanalytical radius of the bead derived for a special case	nm^{-1}
R_0	Radius of the cylinder	nm
l_k	Induced length scale by a rigid protein domain	nm
l_σ	Induced length scale by a protein-induced spontaneous curvature	nm

2.2 Table of parameters

Table 3: Value of parameters used in the model for different figures

Notation	Description	Value
λ_0	Figs. 3, 4, 6A, 6B, 7, S4, S3, S5, S7, and S9	$0.064\ \mathrm{pN}\cdot\mathrm{nm}^{-1}$
	Figs. 6E and 6F,	$0.004 – 0.064\ \mathrm{pN}\cdot\mathrm{nm}^{-1}$
	Figs. S1,	$0 – 0.064\ \mathrm{pN}\cdot\mathrm{nm}^{-1}$
κ_{ratio}	Figs. 3, 6A, 7A,7E, 7F, S1, S3,S2, and S5	1
	Figs. 4 and S4	1 – 30
	Figs. 6A and S9A	11
	Figs. 6B , 6E, 7F, and S9B	1 – 11
	Fig. 7E	4
	Fig. S7	5
σ_0	Figs. 3, S3, and S2	$0 – 1.25\times10^{-4}\ \mathrm{nm}^{-2}$
	Figs. 6B, 6E, 7E, 7H, S7, and S9B	$0 – 3.75\times10^{-4}\ \mathrm{nm}^{-2}$
	Figs. 4, 7B, S4, and S5	$1.25\times10^{-4}\ \mathrm{nm}^{-2}$
	Figs. 7D	$8.25\times10^{-5}\ \mathrm{nm}^{-2}$
	Figs.7F and 7I	$3\times10^{-4}\ \mathrm{nm}^{-2}$
	Fig. 7G	$1.875\times10^{-5}\ \mathrm{nm}^{-2}$
α	Constant for all figures except Fig. S2	$128\times10^{5}\ \mathrm{nm}^3\cdot\mathrm{pN}$
	Fig. S2	$(12.8\text{-}128\text{-}1280)\times10^{5}\ \mathrm{pN}\cdot\mathrm{nm}^3$
β	Constant for all figures	$512\times10^{9}\ \mathrm{pN}\cdot\mathrm{nm}^5$
$\mu\varphi$	Constant for all figures	-200 nm
L_{rigid}	Constant for all figures	$8\ \mu\mathrm{m}$
L_{protein}	Constant for all figures	$8\ \mu\mathrm{m}$
$L_{\text{separation}}$	Constant for all figures	$4\ \mu\mathrm{m}$
p	For all figures	0

3 Supplementary Figures

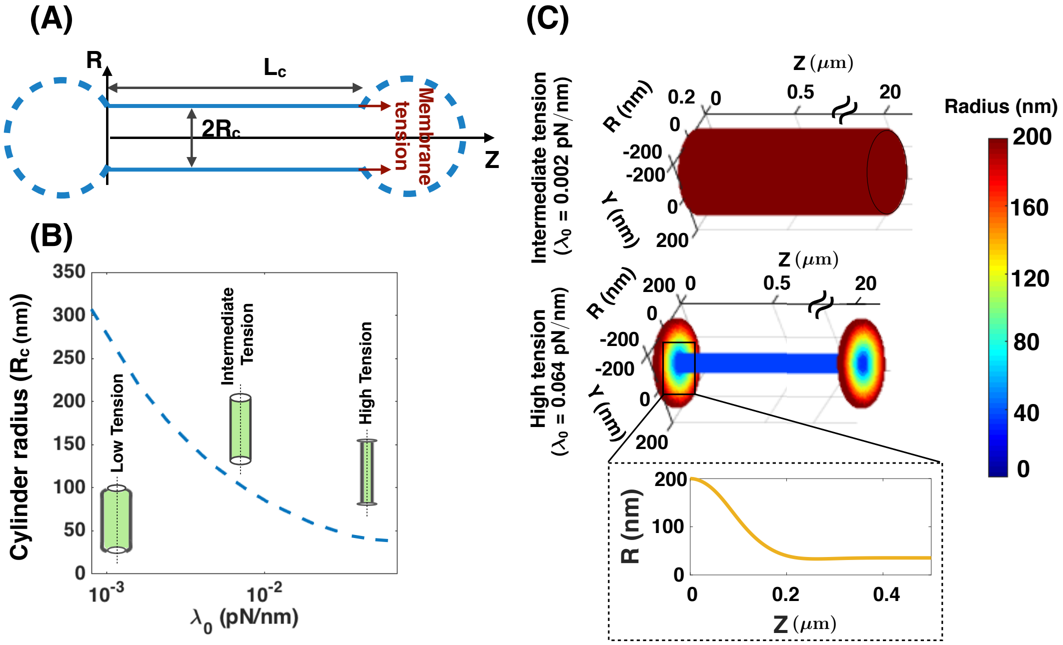

Figure S1: Tension-mediated nanotube formation. (A) Schematic showing the simplest morphology for a nanotube, a cylinder with a radius R_c and length $L_c = 100R_c$ between two adjacent cells in the absence of both membrane cytoskeleton and osmotic pressure. (B) Tubular membrane becomes narrower by tuning the membrane tension at the boundary. Tube radius is plotted as a function of applied tension at the boundary (λ_0). With increasing edge tension, the cylinder radius (R_c) decreases. Based on the ratio of the length and radius, the observed shapes can be classified in three categories: (1) a wide cylinder ($L_c/R_c < 100$) at low tension, (2) a perfect cylinder ($L_c/R_c = 100$) at intermediate tension, and (3) a narrow cylinder similar to a nanotube ($L_c/R_c > 100$) at high tension. (C) Radius variation in a tubular membrane versus the narrow nanotube. A perfect cylinder stabilized at an intermediate tension ($\lambda_0 = \kappa_{\text{lipid}}/4R_c^2 = 0.002\,\text{pN/nm}$) has a uniform radius ($R_c = 200\,\text{nm}$). In contrast, the narrow nanotube at high tension ($\lambda_0 = 0.064\,\text{pN/nm}$), has large radii at the edges ($R_c = 200\,\text{nm}$) and a small radius along the connecting tubule ($R_c = 40\,\text{nm}$). The inset shows the smooth transition of the nanotube morphology at the edge boundary.

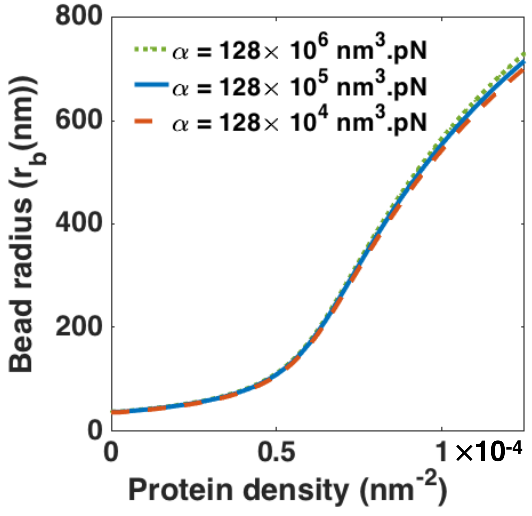

Figure S2: Bead radius as a function of the protein density for three different values of protein aggregation strength (α). There is no significant change in the bead radius and morphology with varying α by two orders of magnitude.

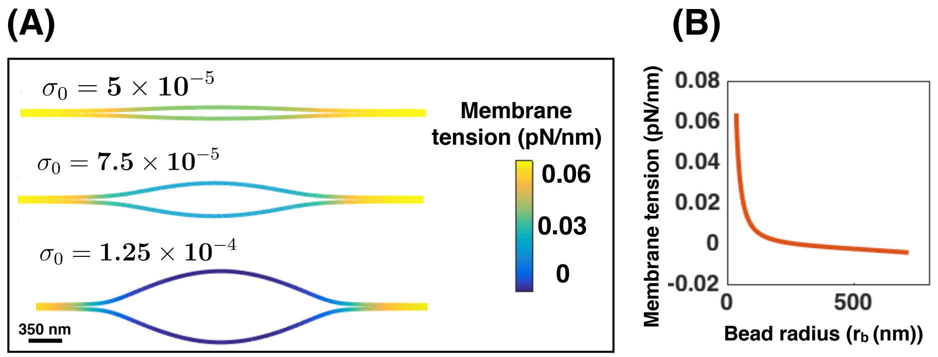

Figure S3: Membrane tension distribution along nanotubes corresponding to local protein aggregation and shown in Fig. 3. (A) The region of the protein aggregation and bead-shaped deformations have a lower membrane tension compared to the rest of the membrane. (B) Reduction of the local membrane tension along the beading domain versus the bead radius. Here the negative membrane tension can be interpreted as a surface pressure [5].

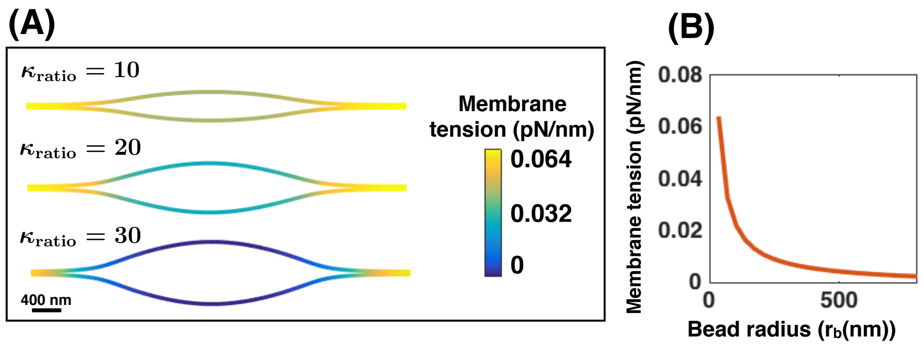

Figure S4: Membrane tension distribution along nanotubes corresponding to local bending rigidity variation shown in Fig. 4. (A) The region of higher rigidity and bead-shaped deformations have a lower membrane tension compared to the rest of the membrane. (B) Reduction of the local membrane tension along the beading domain versus the bead radius.

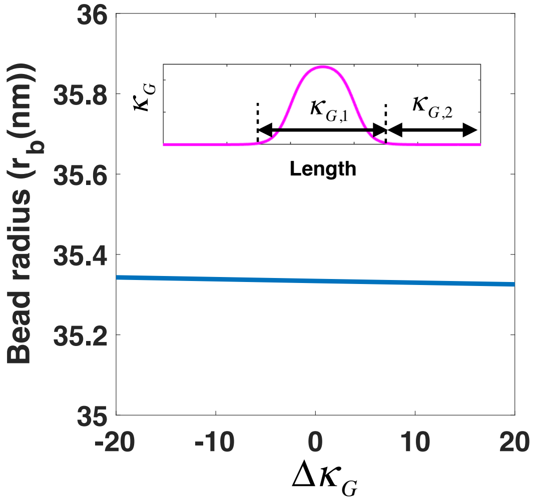

Figure S5: Beads do not form with a localized Gaussian modulus variation. Here we applied a variable Gaussian modulus splay – prescribed as a hyperbolic tangent Eq. (S33) – along a constant length of the membrane ($L_{\text{Gaussian}} = 8\ \mu\text{m}$) at the center of the nanotube, $\lambda_0 = 0.064\,\text{pN/nm}$. We define $\Delta\kappa_{\text{G}} = (\kappa_{\text{G},1} - \kappa_{\text{G},2})/\kappa_{\text{lipid}}$ varying between negative and positive values [20, 22]. There is a negligible membrane deformation with increasing the Gaussian moduli difference from $\Delta\kappa_{\text{G}} = -20$ to $\Delta\kappa_{\text{G}} = 20$ such that the bead radius is almost equal to the nanotube radius ($R_c = 35.334\,\text{nm}$) at $\Delta\kappa_{\text{G}} = 0$.

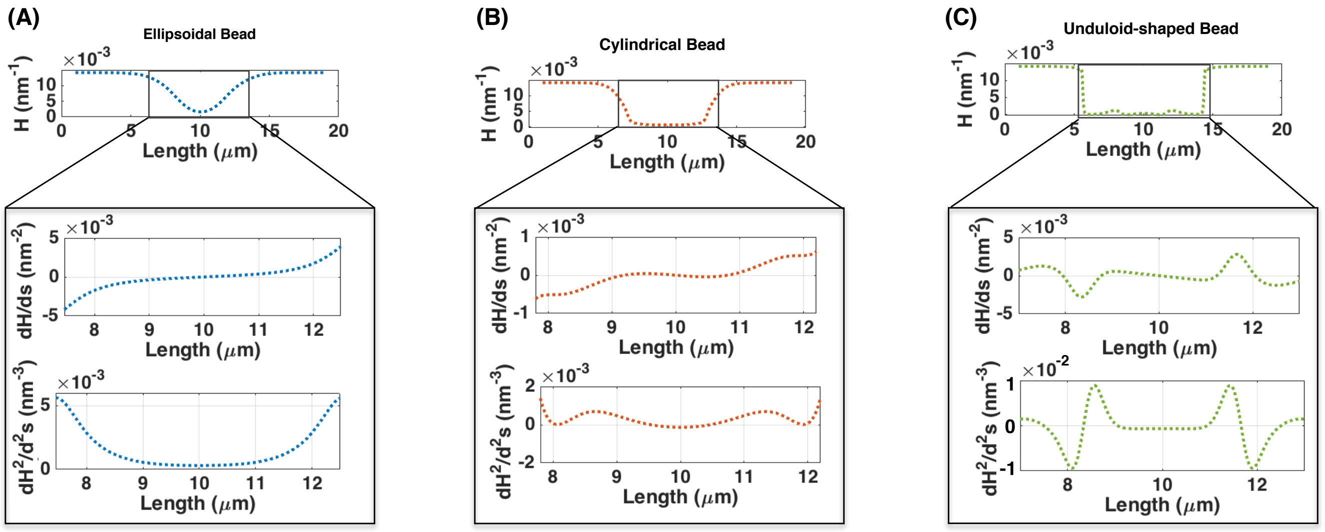

Figure S6: Classification of three different beads based on the second derivative of the mean curvature (H'') along the protein-enriched domain.(A, upper) Mean curvature (H) along the ellipsoidal bead. (A, lower) H' changes sign from negative to positive along the ellipsoidal bead but H'' is always positive. (B, upper) H distribution along the cylindrical bead. (B, lower) Both H' and H'' are smaller compared to the ellipsoidal bead and their sign change along the bead. (C, upper) H distribution along the unduloid-shaped bead. (C, lower) The sign of both H' and H'' changes along the bead.

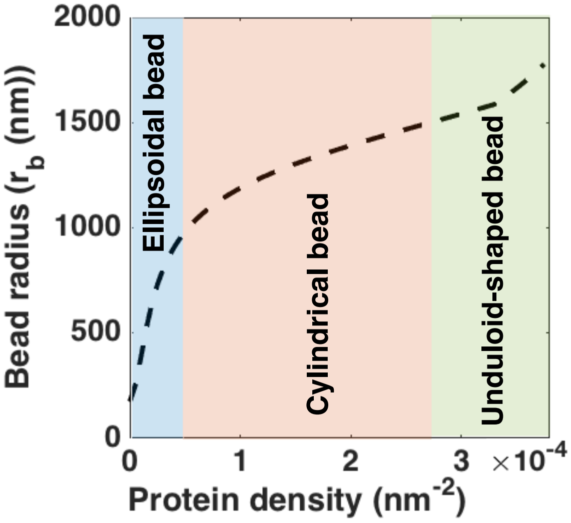

Figure S7: Bead radius as a function of the protein density for a rigid protein-enriched domain ($\kappa_{\text{ratio}} = 5$). The three different observed bead morphologies in Fig. 6 are separated with the same colors. The slope of the bead radius versus the protein density in cylindrical and unduloid-shaped beads is smaller compared to the ellipsoidal bead.

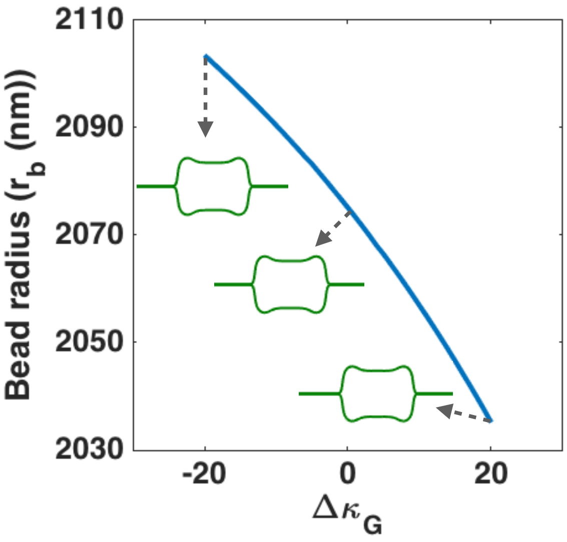

Figure S8: Decrease in the radius of the undoid-shaped bead with increasing the Gaussian modulus $\Delta\kappa_G$ from negative to positive values, $\kappa_{\text{ratio}} = 11$ and $\sigma_0 = 1.85 \times 10^{-4}\ \text{nm}^{-2}$. While the heterogeneity in the Gaussian modulus alters the radius of the bead, it does not affect the morphology of the bead, the undoloid-shape bead remains as a unduloid. Also, the change in the radius of the bead is small.

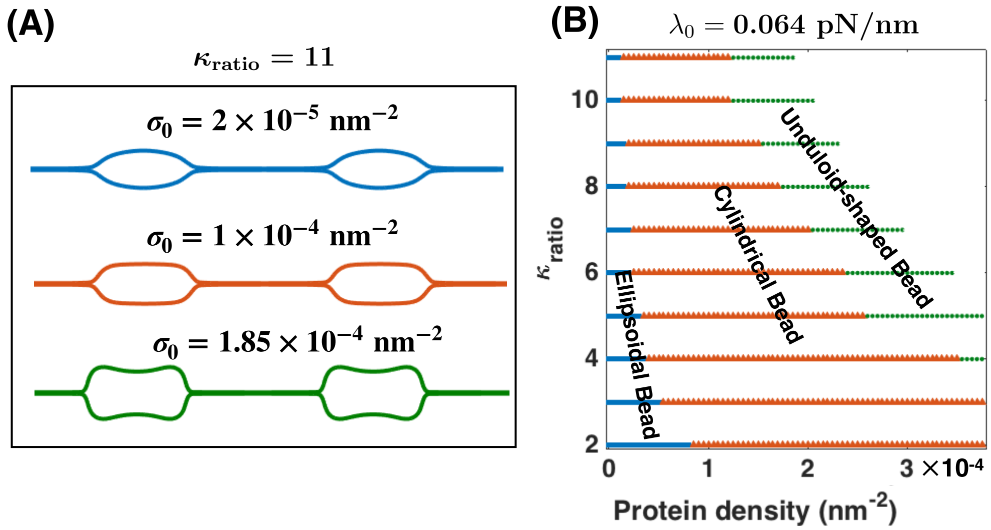

Figure S9: Formation of two beads far away from each other with no interaction. (A) Three different shapes of two beads in the presence of two rigid domains of proteins with $\kappa_{\text{ratio}} = 11$, and $\lambda_0 = 0.064\,\text{pN/nm}$. Here, we set the protein density distribution the same as Fig. 7A. Similar to a single bead (Fig. 6A), as protein density increases, each bead shape transforms independently from an ellipsoidal bead to a cylindrical one and finally a unduloid-shaped bead. (B) The bending rigidity ratio versus the number of the proteins per unit area phase diagram for $\lambda_0 = 0.064\,\text{pN/nm}$. As expected, the phase diagram is exactly the same as Fig. 6A because the beads are far away from each other and therefore their shapes evolve completely independently of one another.

References

1. D. Steigmann, “Fluid films with curvature elasticity,” *Archive for Rational Mechanics and Analysis*, vol. 150, no. 2, pp. 127–152, 1999.

2. D. Steigmann, E. Baesu, R. E. Rudd, J. Belak, and M. McElfresh, “On the variational theory of cell-membrane equilibria,” *Interfaces and Free Boundaries*, vol. 5, no. 4, pp. 357–366, 2003.

3. D. J. Steigmann, “Mechanics and physics of lipid bilayers,” in *The Role of Mechanics in the Study of Lipid Bilayers*, pp. 1–61, Springer, 2018.

4. P. Rangamani, A. Agrawal, K. K. Mandadapu, G. Oster, and D. J. Steigmann, “Interaction between surface shape and intra-surface viscous flow on lipid membranes,” *Biomechanics and modeling in mechanobiology*, pp. 1–13, 2013.

5. P. Rangamani, K. K. Mandadap, and G. Oster, “Protein-induced membrane curvature alters local membrane tension,” *Biophysical journal*, vol. 107, no. 3, pp. 751–762, 2014.

6. H. Alimohamadi, R. Vasan, J. Hassinger, J. Stachowiak, and P. Rangamani, “The role of traction in membrane curvature generation,” *Biophys. J.*, vol. 114, no. 3, p. 600a, 2018.

7. N. Gov, “Guided by curvature: shaping cells by coupling curved membrane proteins and cytoskeletal forces,” *Phil. Trans. R. Soc. B*, vol. 373, no. 1747, p. 20170115, 2018.

8. A. Veksler and N. S. Gov, “Phase transitions of the coupled membrane-cytoskeleton modify cellular shape,” *Biophysical journal*, vol. 93, no. 11, pp. 3798–3810, 2007.

9. S. Leibler and D. Andelman, “Ordered and curved meso-structures in membranes and amphiphilic films,” *Journal de physique*, vol. 48, no. 11, pp. 2013–2018, 1987.

10. H. Hagerstrand, L. Mrowczynska, U. Salzer, R. Prohaska, K. A. Michelsen, V. Kralj-Iglic, and A. Iglic, “Curvature-dependent lateral distribution of raft markers in the human erythrocyte membrane,” *Mol. Membr. Biol.*, vol. 23, no. 3, pp. 277–288, 2006.

11. D. Andelman, T. Kawakatsu, and K. Kawasaki, “Equilibrium shape of two-component unilamellar membranes and vesicles,” *EPL (Europhysics Letters)*, vol. 19, no. 1, p. 57, 1992.

12. Z. Shi and T. Baumgart, “Dynamics and instabilities of lipid bilayer membrane shapes,” *Advances in colloid and interface science*, vol. 208, pp. 76–88, 2014.

13. J. W. Cahn and J. E. Hilliard, “Free energy of a nonuniform system. i. interfacial free energy,” *The Journal of chemical physics*, vol. 28, no. 2, pp. 258–267, 1958.

14. R. Dimova, “Recent developments in the field of bending rigidity measurements on membranes,” *Advances in colloid and interface science*, vol. 208, pp. 225–234, 2014.

15. W. Helfrich, “Elastic properties of lipid bilayers: theory and possible experiments,” *Zeitschrift fur Naturforschung C*, vol. 28, no. 11-12, pp. 693–703, 1973.

16. A. Agrawal and D. J. Steigmann, “A model for surface diffusion of trans-membrane proteins on lipid bilayers,” *Zeitschrift fur Angewandte Mathematik und Physik (ZAMP)*, vol. 62, no. 3, pp. 549–563, 2011.

17. M. Chabanon and P. Rangamani, “Gaussian curvature directs the distribution of spontaneous curvature on bilayer membrane necks,” *Soft Matter*, 2018.

18. A. Agrawal and D. J. Steigmann, “Modeling protein-mediated morphology in biomembranes,” *Biomechanics and modeling in mechanobiology*, vol. 8, no. 5, pp. 371–379, 2009.

19. A. Agrawal and D. J. Steigmann, “Boundary-value problems in the theory of lipid membranes,” *Continuum Mechanics and Thermodynamics*, vol. 21, no. 1, pp. 57–82, 2009.

20. J. E. Hassinger, G. Oster, D. G. Drubin, and P. Rangamani, “Design principles for robust vesiculation in clathrin-mediated endocytosis,” *Proceedings of the National Academy of Sciences*, vol. 114, no. 7, pp. E1118–E1127, 2017.

21. H. Alimohamadi, R. Vasan, J. Hassinger, J. C. Stachowiak, and P. Rangamani, “The role of traction in membrane curvature generation,” *Mol. Biol. Cell*, vol. 29, no. 16, pp. 2024–2035, 2018.

22. T. Baumgart, S. Das, W. Webb, and J. Jenkins, “Membrane elasticity in giant vesicles with fluid phase coexistence,” *Biophysical journal*, vol. 89, no. 2, pp. 1067–1080, 2005.
